# Tomato SlAN11 regulates flavonoid biosynthesis and seed dormancy by interaction with bHLH proteins but not with MYB proteins

**DOI:** 10.1038/s41438-018-0032-3

**Published:** 2018-06-01

**Authors:** Yongfeng Gao, Jikai Liu, Yongfu Chen, Hai Tang, Yang Wang, Yongmei He, Yongbin Ou, Xiaochun Sun, Songhu Wang, Yinan Yao

**Affiliations:** 1 School of Life Science and Engineering, Southwest University of Science and Technology, Mianyang, 621010 China; 2 Yunnan Engineering Laboratory for Agro-environment Pollution Control and Eco-remediation, The Innovation Team for Farmland Non-pollution Production of Yunnan Province, Kunming, 650201 China; 3 Shaanxi Collaborative Innovation Center of Chinese Medicinal Resources Industrialization, Shaanxi University of Chinese Medicine, Xianyang, 712046 China; 4 Chengdu Institute of Biology, Chinese Academy of Sciences, Chengdu, 610041 China

## Abstract

The flavonoid compounds are important secondary metabolites with versatile human nutritive benefits and fulfill a multitude of functions during plant growth and development. The abundance of different flavonoid compounds are finely tuned with species-specific pattern by a ternary MBW complex, which consists of a MYB, a bHLH, and a WD40 protein, but the essential role of SlAN11, which is a WD40 protein, is not fully understood in tomato until now. In this study, a tomato WD40 protein named as SlAN11 was characterized as an effective transcription regulator to promote plant anthocyanin and seed proanthocyanidin (PA) contents, with late flavonoid biosynthetic genes activated in *35S::SlAN11* transgenic lines, while the dihydroflavonol flow to the accumulation of flavonols or their glycosylated derivatives was reduced by repressing the expression of *SlFLS* in this *SlAN11*-overexpressed lines. The above changes were reversed in *35S::SlAN11-RNAi* transgenic lines except remained levels of flavonol compounds and *SlFLS* expression. Interestingly, our data revealed that *SlAN11* gene could affect seed dormancy by regulating the expressions of abscisic acid (ABA) signaling-related genes *SlABI3* and *SlABI5*, and the sensitivity to ABA treatment in seed germination is conversely changed by *SlAN11*-overexpressed or -downregulated lines. Yeast two-hybrid assays demonstrated that SlAN11 interacted with bHLH but not with MYB proteins in the ternary MBW complex, whereas bHLH interacted with MYB in tomato. Our results indicated that low level of anthocyanins in tomato fruits, with low expression of bHLH (*SlTT8*) and MYB (*SlANT1* and *SlAN2*) genes, remain unchanged upon modification of *SlAN11* gene alone in the transgenic lines. These results suggest that the tomato WD40 protein SlAN11, coordinating with bHLH and MYB proteins, plays a crucial role in the fine adjustment of the flavonoid biosynthesis and seed dormancy in tomato.

## Tomatoes: a protein control for flavonoids

Insights into the production of important nutrients called flavonoids are revealed by a molecular and genetic study of tomatoes. Flavonoids are a diverse group of chemicals with a variety of metabolic functions, and which contribute to the characteristic colors of many fruits and vegetables. They have been linked with health benefits due to their antioxidant, anti-inflammatory, and immune-regulating activities. Researchers in China, led by Yinan Yao at Southwest University of Science and Technology, explored the activity of a protein known as  SlAN11. They found that the protein interacts with other proteins to regulate the activity of specific genes, increasing the production of flavonoids called anthocyanins. SlAN11 also regulates the dormancy process in tomato seeds. Geneticists may use the results to generate new breeds of tomato plants enriched in flavonoids to promote good health.

## Introduction

The flavonoid compounds, a class of secondary polyphenolic metabolites synthesized via the phenylpropanoid pathway, fulfill many vital biological functions and mainly include flavonols or their glycosylated derivatives, anthocyanins, and proanthocyanidins (PAs) in the vegetables^1^. Besides producing flower color and providing colors attractive to plant pollinators, flavonoid compounds also play significant roles in ultraviolet-B protection, disease resistance, and plant–microbe interactions, and make possible nutritional and medicinal contribution to human health^2^.

**Fig. 1:**
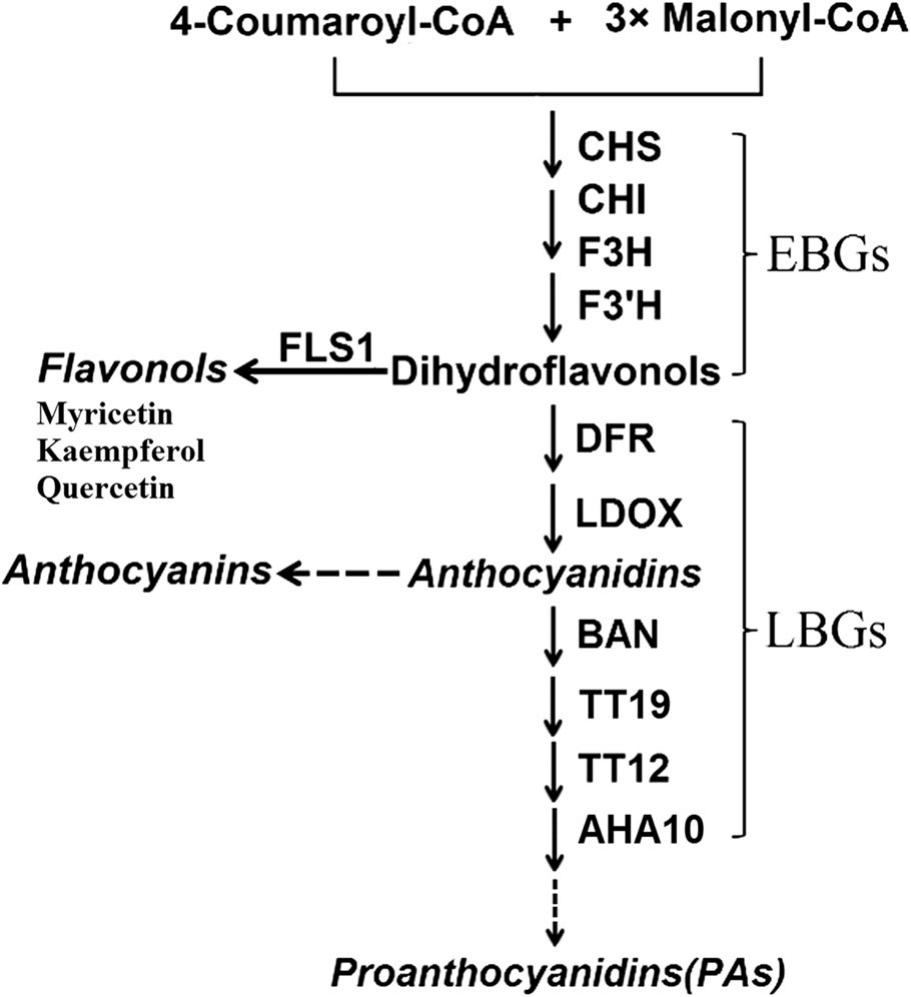
Simplified flavonoid biosynthetic pathway . First, chalcone synthase (CHS) catalyzes the condensation of one molecule of 4-coumaroyl-CoA with three molecules of malonyl-CoA. The later steps in this pathway are catalyzed by a series of enzymes, leading to the production of three main types of final products: flavonols (including quercetin, kaempferol, and myricetin); anthocyanins; and proanthocyanidins (PAs). CHS chalcone synthase, CHI chalcone isomerase, F3H flavanone 3-hydroxylase, F3’H flavonoid 3’-hydroxylase, DFR dihydroflavanol 4-reductase, FLS flavonol synthase, LDOX leucoanthocyanidin dioxygenase, BAN anthocyanidin reductase, TT19 glutathione-*S*-transferase, TT12 multidrug and toxic efflux transporter, AHA10 H^+^ ATPase

The genes encoding the flavonoid biosynthetic enzymes as well as transcriptional factors that regulate the expression of these genes have been identified and characterized from petunia, *Arabidopsis*, snapdragon, maize, and other plant species^2,3^. It plays a vital role in the spatial and temporal expression of structural genes in flavonoid biosynthesis by forming a ternary MBW complex composed of an R2R3-MYB protein, a protein with basic helix-loop-helix (bHLH) domain and a Trp-Asp (WD)-repeat protein. Based on whether be regulated by the MBW protein complex, the genes encoding common enzymes shared by flavonoid biosynthesis pathways can be subdivided into late biosynthetic genes (LBGs; e.g., *DFR*, *LDOX*, *BAN*, *TT19*, *TT12*, and *AHA10*) that are dependent on this complex, and early biosynthetic genes (EBGs; e.g., *CHS*, *CHI*, *F3H*, *F3’H*, and *FLS1*) that are not (Fig. 1)^4–6^.

WD proteins are defined by the presence of four or more highly conserved repeating units usually terminating in a WD dipeptide, and belong to a huge family existed in all eukaryotes^7^. To date, members of this family are involved in many functions like signal transduction, transcription regulation, and cell cycle regulation^8^. The first WD40 gene that functions in regulating anthocyanins biosynthesis was identified in petunia by transposon tagging means and named *Anthocyanins11* (*AN11*), which regulates the pigmentation of the flower^9^. Subsequently, the *Transparent Testa Glabra1* (*TTG1*) locus in *Arabidopsis* was isolated and was found to encode a WD40 repeat protein^10^. Previous studies have shown that *TTG1* is expressed in all examined tissues of tested plants, and plays various roles in *Arabidopsis*, including anthocyanin and PA biosynthesis, seed coat mucilage, trichome, and root hair patterning^11^. To date, homologs of *TTG1* have been identified and shown species-specific function in different plant species, like *ZmPAC1* from *Zea mays*, *MtWD40-1* in *Medicago*, *FaTTG1* in strawberry, and *AaTTG1* from *Arabis alpine*^12–15^.

As an economically important crop, tomato (*Solanum lycopersicum*) provide the largest dietary source of lycopene and other carotenoids, like β-carotene, which serve as an important bioactive compound beneficial to human health^16^. However, in most of the tomato varieties, flavonoids/anthocyanins are generally not present in both tomato fruit peel and flesh tissues^17^. Recently, several members of MBW complex in tomato have been identified and partially characterized as a regulator of anthocyanin biosynthesis. Two paralog genes encoding homologous R2R3-MYB transcription factors (TFs), *Anthocyanins1* (*SlANT1*) and *Anthocyanins2* (*SlAN2*), are both mapped on chromosome 10 of tomato and highly homologous with *PhAN2* in *Petunia x hybrid*^18,19^. Ectopic expression of *SlANT1* and *SlAN2* can induce anthocyanin biosynthesis in the various organs of the transgenic tomato lines, and SlAN2 mediated the trigger of anthocyanin biosynthesis induced by high light as well as cold in vegetative organs^20^. *SlGL3* gene, which encodes a bHLH TF homologous to *Arabidopsis* GLABRA3 (GL3), could repress anthocyanin accumulation when heterologously expressed in *Arabidopsis*, suggesting that SlGL3 functions as a repressor of anthocyanins accumulation^21^. More recently, overexpression of another bHLH TF gene wild-type (WT) *AH* in FMTT271 (a tomato inbred line contains a mutated allele of *AH*, which showed no anthocyanin pigmentation) led to higher anthocyanin accumulation and improved transcript of several anthocyanin biosynthesis genes, demonstrating that *AH* serves as a key transcriptional regulator of anthocyanin biosynthesis in *S. lycopersicum*^22^.

Despite much knowledge of the MBW complex model regulating the flavondoid/anthocyanin biosynthesis in many plant species, most of the identified genes belong to the R2R3-MYB and bHLH TF family in tomato until now, little is known about the roles of WD-repeat proteins in regulation of flavonoid/anthocyanin biosynthesis and other developmental processes in tomato. In addition, the relative contributions of bHLH, MYB, and WD-repeat protein to the flavonoid/anthocyanin pigment accumulation and its tissue-specific patterns in tomato remain poorly described. In this study, we characterized the molecular function of a tomato WD40 gene, named *SlAN11* (for homology to *PhAN11*)^20^, and demonstrated that it participated in flavonoid biosynthesis in both tomato plant and seeds. In addition, we examined its interactions with bHLH and MYB TFs, and their transcriptional activities by yeast two (Y2H)-/one-hybrid assays. Moreover, our data revealed a novel role of *SlAN11* gene in controlling seed dormancy as well as its relation with ABA signaling. Finally, the deficiency of gene transcripts of *SlANT1*, *SlAN2*, and *SlTT8* may shed some light on the absence of anthocyanin accumulation in tomato fruits.

## Materials and methods

### Phylogenetic analysis

SlAN11 and homologous WD40 proteins were identified from the NCBI Network using the blastp search tool in the reference proteins database. WD-repeat motif sequences of these proteins were defined using the protein prediction program available at the European Bioinformatics Institute (http://www.ebi.ac.uk/interpro/). To analyze the phylogenetic relationship between SlAN11 and WD40 proteins from 16 other species, full-length amino-acid sequences were aligned by MEGA 6. An unrooted tree was constructed using the neighbor-Joining method, with 1000 bootstrap value.

### Material and plant growth conditions

Tomato (*S. lycopersicum*) cv Ailsa Craig (LA2838A) was obtained from the TGRC (http://tgrc.ucdavis.edu/). The WT tomato plants and transgenic plants were grown in the greenhouse under long-day conditions (26 °C day, 18 °C night; 16 h light, 8 h dark), with a relative humidity of 50–60%. Primary transformants (T_0_) and their offsprings were planted under the long-day conditions and then transplanted into the field 4–6 weeks later.

### Plasmid construction and tomato transformation

DNA manipulations were performed by using standard molecular biology techniques^23^. In order to generate the overexpression constructs, *SlAN11* (accession No. XM_004235284.3) were amplified from cDNAs by PCR using specific primers (Supplementary Table 1). The 1029 bp fragment obtained by PCR ligated to plant expression vector pBI121 under the transcriptional control of the 35SCaMV promoter. The resulting construct pBI121-*35S*_*Pro*_*::SlAN11* was created.

Sequences from *SlAN11* cDNA were amplified by PCR for construction of the 35S::SlAN11-RNAi vector. An inverted-repeat target gene fragment was constructed in vector pSKint^24^ and transferred into pBI121 (driven by the 35S promoter) at the *Xba*I and *Sac*I restriction sites by PCR using specific primers (Supplementary Table 1). The resulting construct pBI121-*35S*_*Pro*_*::SlAN11-RNAi* was created.

For determining the subcellular localization of SlAN11 protein, the complete open reading frame (ORF) without the stop codon of *SlAN11* was amplified by using primers (Supplementary Table 1), incorporating restriction sites *Xba*I and *Xho*I at the primer ends. The amplified fragments were cloned into the expression vector pTEX-GFP to generate pTEX-SlAN11-GFP.

To isolate *SlAN11* promoter from tomato genomic DNA, PCR specific primers were designed (Supplementary Table 1). The location of forward primer was approximately 2 kb upstream of the translation start site. A 2034 bp *SlAN11* promoter was amplified by PCR and ligated to pBI121 plasmid vector and fused with *GUS* report gene, replacing the 35SCaMV promoter. Consequently, the *GUS* expression construct pBI121-*SlAN11*_*Pro*_*::GUS* was generated.

Above pBI121-*35S*_*Pro*_*::SlAN11*, pBI121-*35S*_*Pro*_*::SlAN11-RNAi*, and pBI121-*SlAN11*_*Pro*_*::GUS* plasmids were transferred separately to *Agrobacterium tumefaciens* EHA105 and the recombinant strains were used to transform tomato according to the method described by Fillatti et al.^25^. The transformed lines with transgene insertion were first selected for kanamycin (70 mg/L) resistance, and then confirmed by PCR using *NPTII*-specific primers.

### Quantitative real-time PCR assays

Quantitative real-time PCR (qRT-PCR) was performed with the IQ SYBR Green Supermix (Bio-rad catalog #1708882) using the Applied Biosystems Step One Real-Time PCR System, with tomato *SlUBI3* gene as an internal reference. The relative expression values were determined against the WT sample using the 2^−ΔΔCt^ method. All the primers used for qRT-PCR analysis are provided in Supplementary Table 2.

### β-glucuronidase staining assay

Histochemical staining of β-glucuronidase (GUS) activity of transgenic plants harboring *SlAN11*_*Pro*_*::GUS* was performed according to described previously^26^. Stained tissues or organs were visualized using a Leica microscope coupled to an insight digital camera.

### Subcellular localization of SlAN11

The plasmid pTEX-GFP as positive control and pTEX-SlAN11-GFP were separately transfected into tomato mesophyll protoplasts, essentially as described previously^27^. Protoplasts were tested for SlAN11-GFP expression using a confocal microscope at 514 nm wavelength (LSM 5Exciter, Carl-Zeiss) after being incubated at 23 °C for 16–18 h.

### Anthocyanin assays

Anthocyanins were extracted from fully expanded leaves and stems of 30-day-old tomato plants and from mature red fruit pericarps separately, and assayed according to the methods described previously^28^. In brief, approximately 200 mg of tissue samples were ground in liquid nitrogen, and anthocyanins were extracted for 48 h in darkness with shaking in 0.5 mL of 1% (v/v) HCl in methanol. A total of 0.4 mL distilled water and 1 mL of chloroform was added to the samples to separate the anthocyanins, followed by centrifugation for 5 min at 3000 rpm. The upper aqueous phase was determined by measuring the optical density at A535 nm (A535). Anthocyanin content was calculated using the simple formula (A535)/mg fresh weight.

### PA extraction and analysis

PAs of tomato seeds were determined using by Vanillin-HCl method^29^. A unit of 100–200 mg of ground tomato seeds was extracted with 2 mL 60% ethyl alcohol, sonicated for 30 min. The slurry was centrifuged (12 000 ×*g*) at room temperature, and the 200 µL supernatant was transferred to a brown tube which 800 µL of methanol: 4% HCl solution containing 4% vanillin was added. After stirring with a tube mixer, and incubated for 30 min at 25 °C, the absorbance at 500 nm of the red solution was measured by a spectrophotometer. PA content was calculated using the standard curve made by standard PAs (REBIO, R131074).

### Flavonoid staining in seedlings

Seven-day-old seedlings of WT and transgenic tomato seedlings were stained according to previously described method^30^. Seedlings were submerged in aqueous solution containing 0.25% DPBA (w/v) and 0.02% (v/v) Triton X-100 for 5 min, and then washed for 5 min in distilled water. The roots were visualized using a Confocal Laser Scanning Microscope with an Ar-laser (458/488/514 nm) for green fluorescent protein (GFP) and a 543 nm HeNe-laser for yellow fluorescent protein.

### Seed germination assays

The germination assays were performed as described previously^31^. In brief, 100–200 WT and T3 homozygous transgenic seeds of the same maturity were surface sterilized by 75% ethanol for 1 min, 15% NaClO for 15 min, and then washed in sterilized water eight times. Seeds were placed on 1/2 Murashige and Skoog medium plates containing the different concentrations of ABA, respectively. Germinated seeds were counted daily for 10 days.

### Yeast one-/two-hybrid assays

Yeast one-hybrid assays were carried out according to the following operation. The full-length CDS (coding sequence) of tomato *SlAN11*, *SlANT1*, *SlAN2*, *SlGL3*, and *SlTT8* were PCR-amplified using primers (Supplementary Table 3). These PCR fragments were cloned into pEG202 vector to obtain LexA DNA-binding domain fusion bait constructs, respectively. The resulting bait construct was transformed into yeast strain EGY48 by LiAc-mediated transformation, and 2–3 days later these yeast strains were tested on selective plate medium or by β-galactosidase activity assay.

For Y2H assays, the full-length CDS of tomato *SlAN11*, *SlANT1*, *SlAN2*, *SlGL3*, and *SlTT8* were PCR-amplified using primers (Supplementary Table 3). These PCR fragments were cloned into pEG202 (containing DNA-binding domain) or pJG4-5 vector (containing activation domain) to obtain bait or prey constructs. Different combinations of bait and prey constructs were co-transformed into yeast strain EGY48 by LiAc-mediated transformation, and 2–3 days later these yeast strains were tested on selective plate medium or by β-galactosidase activity assay. Yeast one-/two-hybrid assays and β-galactosidase quantitative assays were performed according to the procedures in *Current Protocols in Protein Science, 2001. 19.0.1* and *the Yeast Protocols Handbook PT3024-1*.

## Restults

### The *SlAN11* gene is a homolog of a regulatory gene involved in flavonoid/anthocyanin synthesis

The full-length *SlAN11* gene was about 1436 bp, including an ORF of 1029 bp, encoding 342 amino acids. Comparison between the genomic DNA and the cDNA demonstrated that *SlAN11*-lacked introns within the gene sequence like *AtTTG1* identified from *Arabidopsis* and other *TTG1* homologs^9,10,13,32^.

**Fig. 2:**
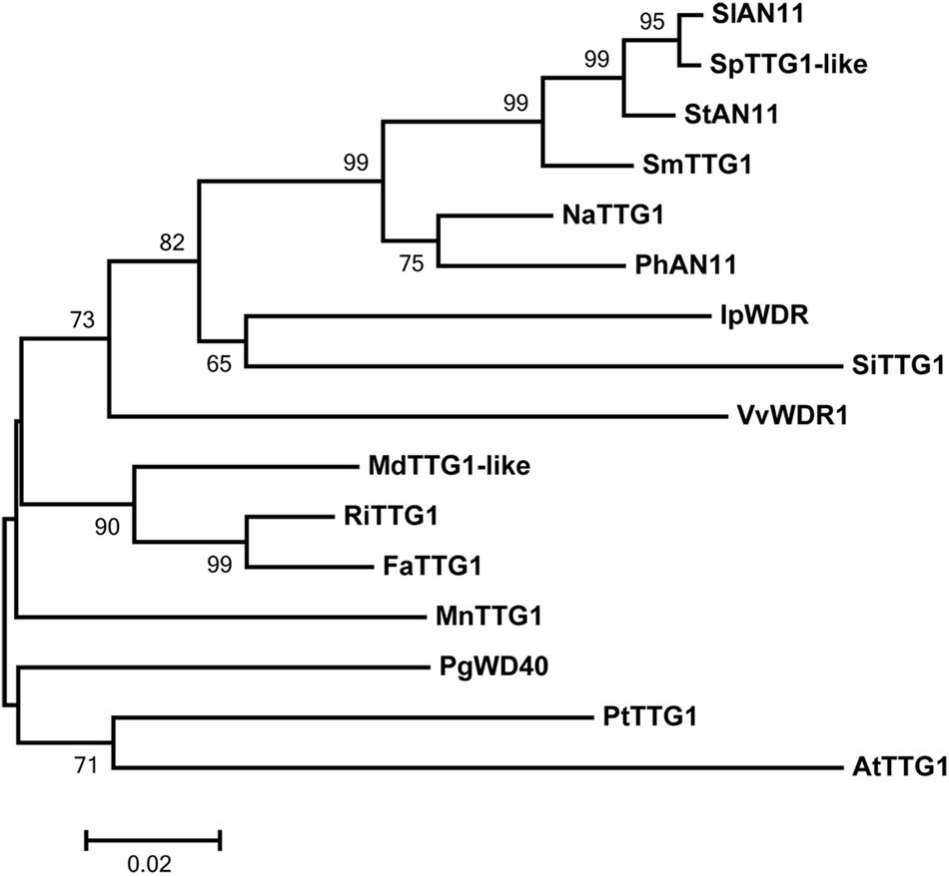
The phylogentic tree of AN11/WD40 protein sequences . The name of the proteins, their source, and GenBank accession numbers refer to figure legend of the Supplementary Fig.S1

At the amino level, the sequence identity between SlAN11 and WD40 protein in other plant species ranged from 77 (AtTTG1) to 99% (SpTTG1-like), with the highest homologs of solanaceous plants, such as 99% (*Solanum pennellii*, SpTTG1-like), 94% (*Nicotiana attenuata*, NaTTG1), and 89% (*Petunia x hybrid*, PhAN11). Lower sequence identity of TTG1 homologs comes from other plant species, for example: 77% (*Arabidopsis thaliana*, AtTTG1); 79% (*Punica granatum*, PgWD40); and 80% (*Populus trichocarpa*, PtTTG1). Phylogenetic analysis of the amino-acid sequences confirmed that SlAN11 clusters together with the proteins from other plants mentioned above (Fig. 2).

All characterized WD40 protein involved in anthocyanins synthesis have four conserved WD-repeat motifs, like AtTTG1, PhAN11, and MdTTG1 as well as VvWDR1, MtWD40-1, PgWD40, etc.^9,10,33^. These four conserved motifs were also found in tomato SlAN11 (Supplementary Figure S1). Moreover, the last two amino-acid residues of these four WD-repeat motif showed high conservation among different species (Supplementary Figure S1), and this was consistent with previous known WD40 proteins related to anthocyanin biosynthesis^9,10,32,33^. The above analysis suggested that the tomato *SlAN11* gene is involved in flavonoid/anthocyanin biosynthesis.

### *SlAN11* is expressed ubiquitously in tomato and SlAN11 protein is targeted to the nucleus

**Fig. 3:**
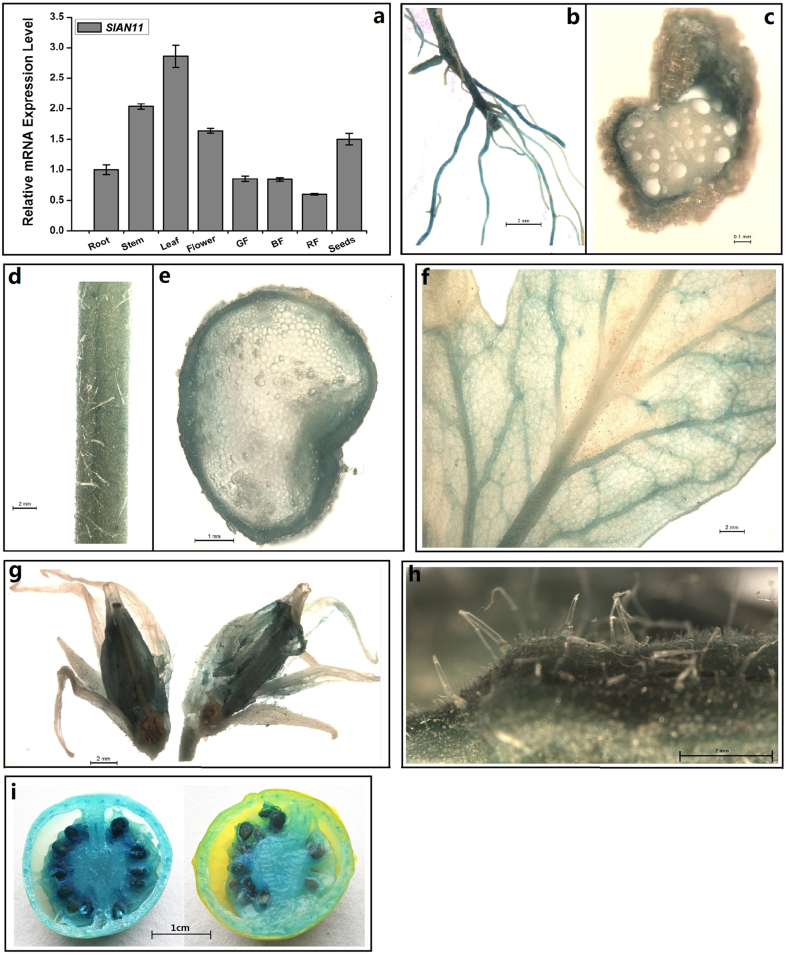
The expression pattern analysis of *SlAN11* in different tissues. **a** The expression levels of *SlAN11* in different tissues. Total RNAs were extracted from roots, stem, leaves, flowers, green fruit pericarps (GF), BF (breaker phase fruit), red fruit pericarps (RF), and mature seeds. The *SlAN11* mRNA levels were quantified by quantitative RT-PCR and are indicated as relative expression levels compared with the internal control *SlUBI3* mRNA. Results represent mean values ± SD from three biological replicates. **b**–**i** Histochemical analysis of GUS staining of *SlAN11*_*Pro*_*::GUS* transgenic plants. The expression of *SlAN11*_*Pro*_*::GUS* transgene was determined by the GUS staining of roots (**b**), transverse sections of roots (**c**), stems (**d**), transverse sections of stems (**e**), leaves (**f**), flowers (**g**), flower buds (**h**), and fruits (**i**)

The transcription of *TTG1* is detected in all major organs of *Arabidopsis*^10^. To study the expression pattern of *SlAN11* gene in tomato, we first examined the expression level of *SlAN11* in roots, stems, leaves, flowers, seeds, and fruit pericarps at different developmental stages of WT plant by using qRT-PCR. As shown in Fig. 3a, *SlAN11* is expressed ubiquitously in all tissues examined, though appears to be highly expressed in leaves, stems, flowers, and seeds.

The expression pattern of *SlAN11* gene was further verified by histochemical GUS reporter assay. To do this, the 2034 bp promoter fragment, including the 5′ untranslated region of *SlAN11* was cloned and fused to a *GUS* reporter gene (*SlAN11*_*Pro*_*::GUS*). Transgenic tomato plants expressing this construct were generated and the *SlAN11*_*Pro*_*::GUS* activity was monitored at different organs. As shown in Fig. 3b–i, GUS staining was detected in all tomato organs, including roots, stems, leaves, flowers (mainly expressed in anther tissue), fruits, and seeds. These results were consistent with the results of qRT-PCR analysis of the tissue expression pattern of *SlAN11* gene (Fig. 3a). Previous studies have shown that TTG1 acts as a trichome-promoting factor to control the epidermal cell fate and trichome patterning in *Arabidopsis*^34,35^. Interestingly, no *SlAN11*_*Pro*_*::GUS* activity was observed in trichome on the stems, leaves, and sepals (Fig. 3d, f, h), indicating that *SlAN11* may not be involved in the control of trichome patterning in tomato.

**Fig. 4:**
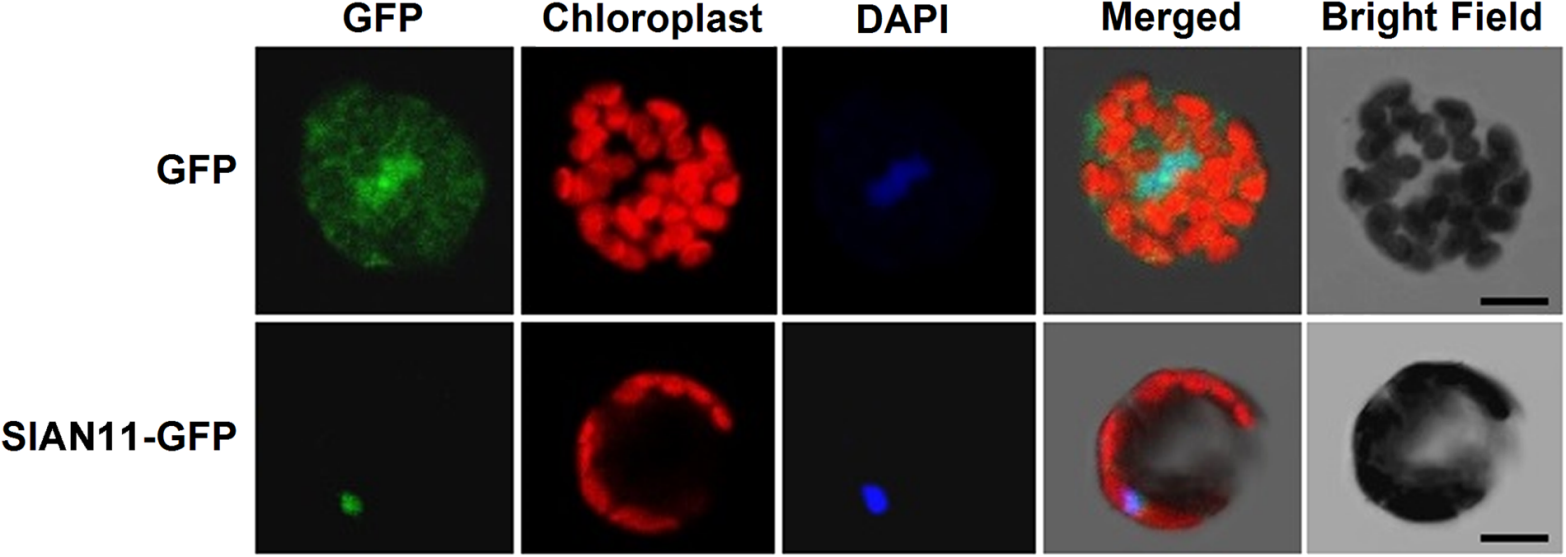
Subcellular localization of SlAN11 protein . The PCR-amplified GFP fragment, encoding green fluorescent protein, was cloned into *Xba*I and *Sal*I sites of pTEX to generate pTEX-GFP. The coding sequences of *SlAN11* genes were PCR-amplified from tomato leaf cDNA and cloned in the pTEX-GFP vector to generate SlAN11-GFP construct. Subcellular localization assay was carried out via transformation of tomato protoplasts. After DAPI staining the protoplasts was examined using confocal microscope to simultaneously capture DAPI and GFP signals. pTEX-GFP construct was included as control. Left to right: green, GFP fluorescence; red, chlorophyll autofluorescence; blue, nucleus stained with DAPI; merged, combined fluorescence from GFP, chlorophyll, and DAPI. Bars, 5 μm

TTG1 and its homologs have been identified as a core member of TTG1/bHLH/MYB transcriptional complex in *Arabidopsis* and other plants species^5,11^, which prompted us to determine the subcellular localization of SlAN11 protein. The GFP was fused to the C terminus of SlAN11 (SlAN11-GFP), and the fusion protein was expressed in tomato mesophyll protoplasts. Compared with the signal of GFP alone, which spread ubiquitously in the protoplasts, the green fluorescent signal of SlAN11-GFP recombinant protein was localized exclusively within the nucleus in the transfected protoplasts (Fig. 4). This observation is consistent with the putative role of SlAN11 that acts as a transcription regulator.

### SlAN11 regulates anthocyanin biosynthesis in tomato plants

**Fig. 5:**
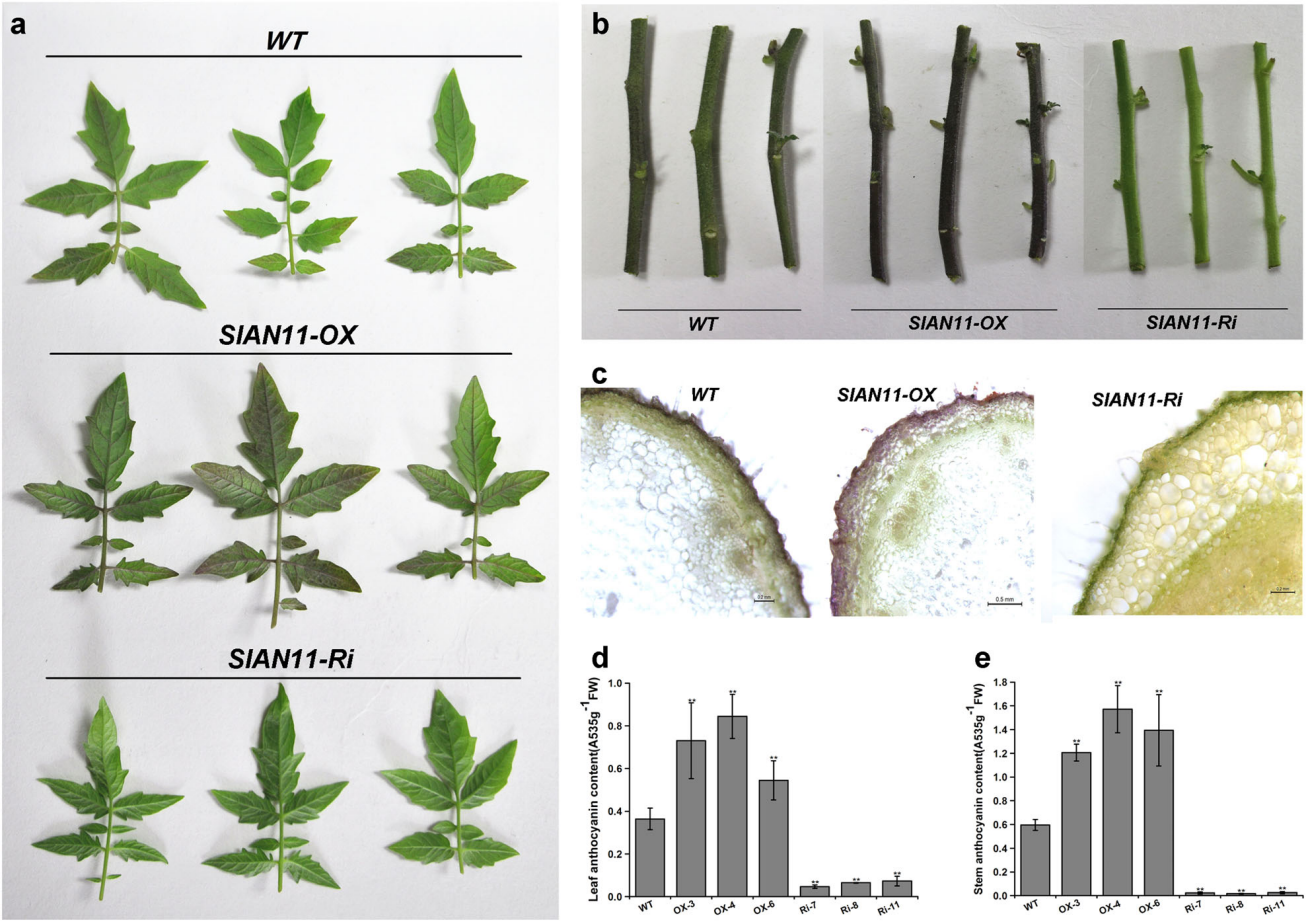
Phenotypes of wild-type and *SlAN11* transgenic plants. **a**, **b** Phenotypes of anthocyanin accumulation in leaves (**a**) and stems (**b**) from field-grown plants of wild-type Ailsa Craig (*WT*), *35S::SlAN11* (*SlAN11-OX*), and *SlAN11-RNAi* (*SlAN11-Ri*) transgenic lines. **c** Microscopic observation of transverse sections of stem from field-grown plants of wild-type, *35S::SlAN11*, and *SlAN11-RNAi* transgenic lines. **d**, **e** Quantitative analysis of anthocyanins contents of leaves (**d**) and stems (**e**) from wild-type, *35S::SlAN11*, and *SlAN11-RNAi* transgenic lines. The plants were grown in greenhouse and harvested 30 days after germination. Results represent mean values ± SD from three biological replicates. Asterisks indicate statistically significant differences (**P < 0.01; *t*-test)

**Fig. 6:**
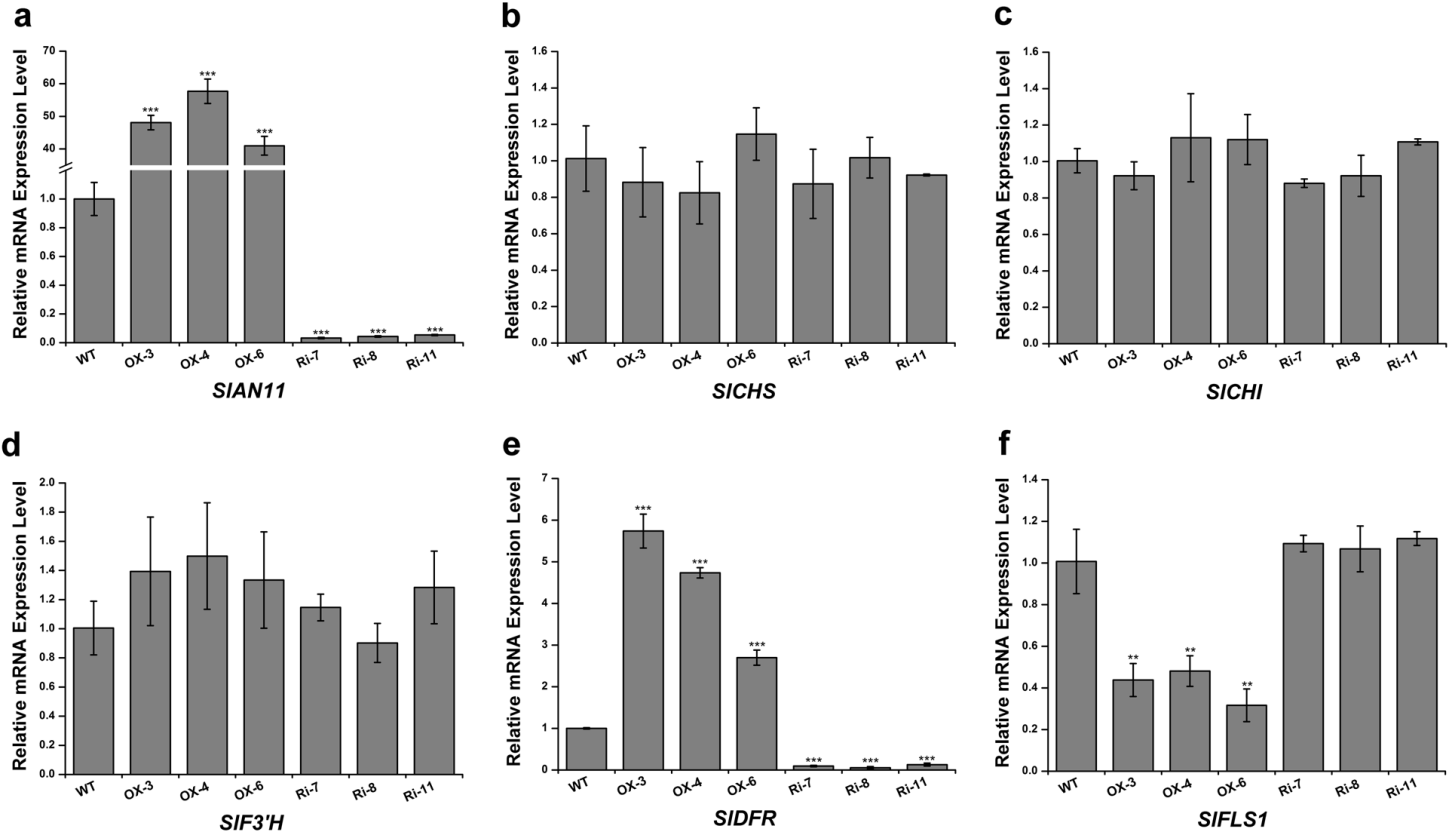
Expression level analysis of genes involved anthocyanin and flavonol biosynthetic pathway in wild-type and *SlAN11* transgenic plants . Total RNA were extracted from seedlings of *WT*, *35S::SlAN11* (lines OX-3, OX-4, and OX-5), and *SlAN11-RNAi* (lines Ri-7, Ri-8, and Ri-11) transgenic plants. Quantitative real-time PCR analysis revealed the relative mRNA levels of these genes, including *SlAN11* (**a**), *SlCHS* (**b**), *SlCHI* (**c**), *SlF3’H* (**d**), *SlDFR* (**e**), and *SlFLS1* (**f**). Results represent mean values ± SD from three biological replicates. Asterisks indicate statistically significant differences (**P < 0.01, ***P < 0.001; *t*-test)

To better understand the functions of *SlAN11*, we generated transgenic tomato plants overexpressing (*35S::SlAN11*) and downregulating (*35S::SlAN11-RNAi*) this tomato gene under the control of the constitutive CaMV 35S promoter. Three independent T_0_ transgenic lines harboring *35S::SlAN11* (OX-3, OX-4, and OX-5) and *35S::SlAN11-RNAi* (Ri-7, Ri-8, and Ri-11) were then randomly selected to generate the T_2_ homozygous tomato plants for further molecular and phenotypic characterization. Compared with WT plants, *SlAN11* overexpression transgenic plants showed enhanced anthocyanin accumulation in leaves and stems, whereas *35S::SlAN11-RNAi* transgenic tomato lines severely lacked anthocyanins in the epidermis and in subepidermal layers of leaves and stems (Fig. 5a–c). Moreover, the 5-day-old transgenic seedlings also have shown enhanced anthocyanin accumulation in the cotyledon and hypocotyl (Supplementary Figure S2). Chemical analysis further confirmed these observations. The overexpression of *SlAN11* increased anthocyanin accumulation by 50–130% and 100–160% in leaves and stems, while anthocyanins content in leaves and stems of *35S::SlAN11-RNAi* transgenic lines was decreased by 5- to 8-fold and 23- to 35-fold, respectively (Fig. 5d). Interestingly, although the anthocyanin production in transgenic plants was significantly altered, no change in trichome development was observed (Supplementary Figure S3), as was consistent with no *SlAN11*_*Pro*_*::GUS* activity in trichome (Fig. 3). To ensure the observed phenotypes correlated with the upregulated and downregulated transcript, the endogenous *SlAN11* mRNA levels in the transgenic plants were determined. Analysis of qRT-PCR revealed a distinct increase and reduction in endogenous *SlAN11* transcript levels in *35S::SlAN11* and *35S::SlAN11-RNAi* lines compared to that of WT plants, respectively (Fig. 6a).

**Fig. 7:**
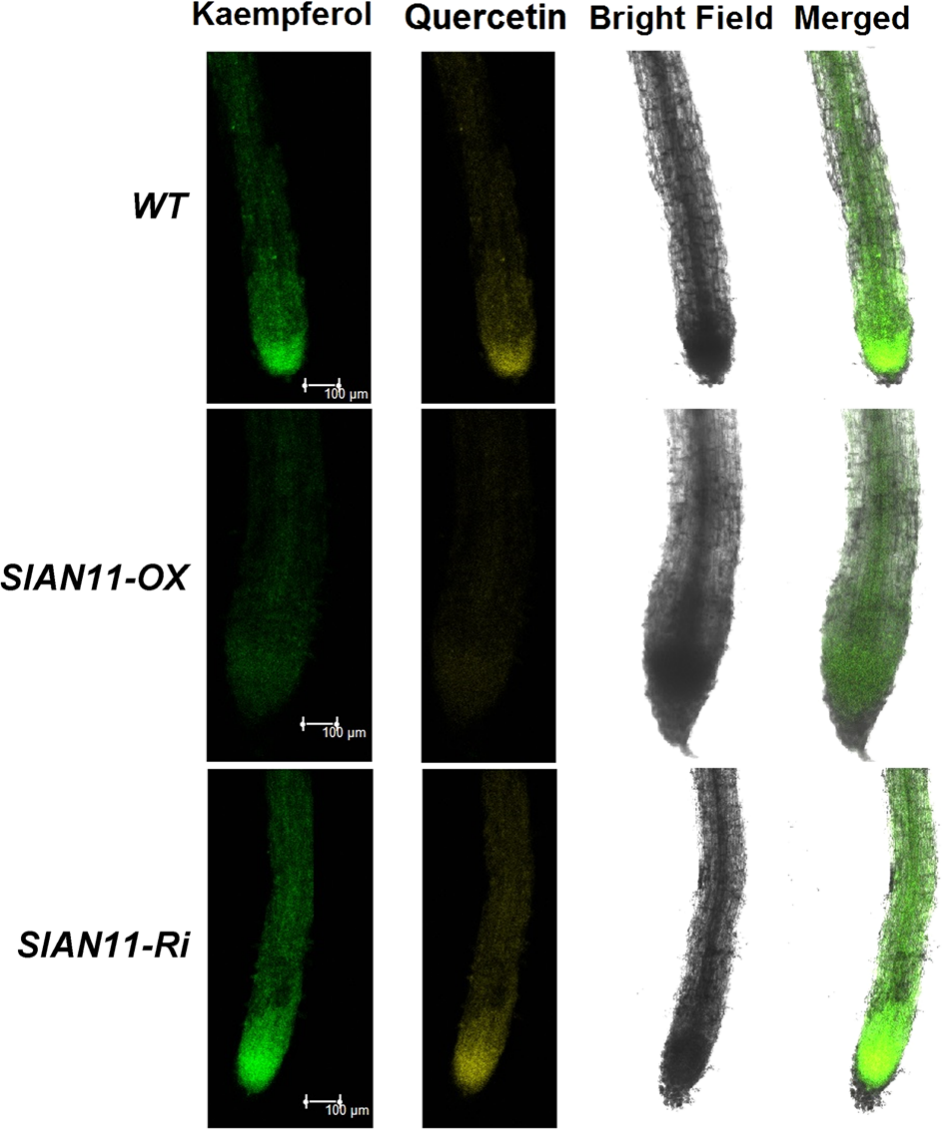
Kaempferol and quercetin (flavonols) accumulation in wild-type and *SlAN11* transgenic seedlings . Seven-day-old wild-type (WT), *35S::SlAN11* (SlAN11-OX), and *SlAN11-RNAi* (SlAN11-Ri) transgenic seedlings grown on 1/2 MS medium were used for DPBA staining for the visualization of kaempferol and quercetin accumulation (bar 100 μm). The roots shown were typical of three separate experiments performed at 23 °C (*n* = 10 seedlings per experiment)

In *Arabidopsis ttg1* mutants, the anthocyanins biosynthetic pathway is blocked at the DFR step, but no effect on transcription of the *CHS* and *CHI* genes was observed^36^. We therefore sought to determine whether the overexpression and downregulation of *SlAN11* gene could change transcriptional levels of genes involved in flavonoid/anthocyanin biosynthetic pathway (Fig. 1). The transcript abundance of EBGs *SlCHS*, *SlCHI*, *SlF3’H*, and *SlFLS* was determined by qRT-PCR analysis as well as the LBGs *SlDFR*. As shown in Fig. 6b–d, expression levels of *SlCHI*, *SlCHS*, and *SlF3’H* genes were not significantly altered in *35S::SlAN11* and *35S::SlAN11-RNAi* transgenic plants compared with WT. The transcript abundance of key anthocyanin synthesis enzyme, *SlDFR*, was dramatically increased and decreased in *35S::SlAN11* and *35S::SlAN11-RNAi* transgenic plants, respectively (Fig. 6e). By contrast, the *SlFLS* transcript was decreased by 50–70% in *35S::SlAN11* transgenic plants, but not significantly changed in *35S::SlAN11-RNAi* transgenic plants (Fig. 6f). As flavonols are synthesized from dihydroflavonols by FLS enzyme, the reduction of *SlFLS* transcript in *35S::SlAN11* transgenic plants prompted us to analyze changes in flavonol accumulation. Seedlings of WT and transgenic lines were stained with DPBA, a dye used to detect flavonols and their glycosylated derivatives visualized using a laser scanning confocal microscopy^30^. As shown in Fig. 7, compared with WT and *35S::SlAN11-RNAi*, the amounts of both K-DPBA (kaempferol) and Q-DPBA (quercetin) fluorescence were significantly decreased in *35S::SlAN11* transgenic seedlings.

### SlAN11 regulates PA biosynthesis in seed coats and seed germination

**Fig. 8:**
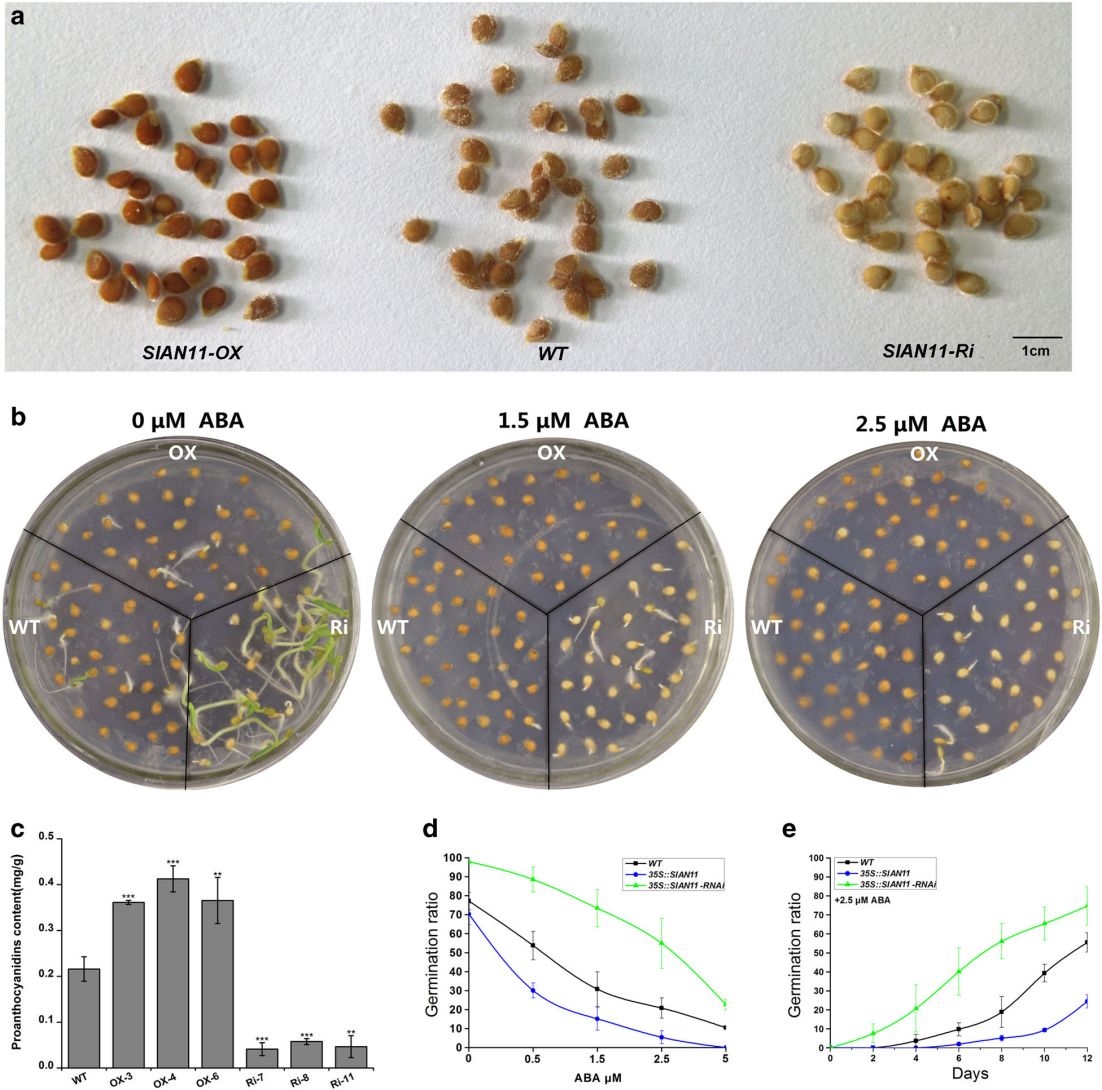
Phenotypes of wild-type and *SlAN11* transgenic seeds. **a** Phenotypes of proanthocyanidin accumulation in seeds from plants of wild-type (*WT*), *35S::SlAN11* (*SlAN11-OX*), and *SlAN11-RNAi* (*SlAN11-Ri*) transgenic lines. **b** Germination phenotype of wild-type (WT), *35S::SlAN11* (*OX*), and *SlAN11-RNAi* (*Ri*) transgenic seeds in the different concentrations of ABA. **c** Quantitative analysis of anthocyanin contents of seeds from wild-type, *35S::SlAN11*, and *SlAN11-RNAi* transgenic lines. Results represent mean values ± SD from three biological replicates. Asterisks indicate statistically significant differences (**P < 0.01, ***P < 0.001; *t*-test). **d** Germination ratio of WT and transgenic T_2_ seeds at the different concentrations of ABA. The percentage of seeds showing root emergence was scored 8 days post stratification. Standard error bars represent three independent experiments. **e** Germination ratio of WT and transgenic T_2_ seeds in 1/2 MS plates with 2.5 μM ABA. Germinated seeds (radicle protruding) were counted daily for 12 days. Germination ratio refers to the number of germinated seeds as a proportion of the total number of seeds. Standard deviation bars represent three independent experiments

To further understand the function of *SlAN11* in seed coat development, we performed a detailed analysis on seed characters of WT and *SlAN11* transgenic lines. As shown in Fig. 8a, compared with WT, overexpression of *SlAN11* gene resulted in enhanced PA accumulation in seed coats, while 35::*SlAN11-RNAi* transgenic seeds exhibited the transparent testa phenotype. Chemical analysis further confirmed these observations. The overexpression of *SlAN11* increased PA accumulation by 60–90% in seeds, while PA content in seeds of *35S::SlAN11-RNAi* transgenic lines was decreased by three- to fivefold (Fig. 8c).

**Fig. 9:**
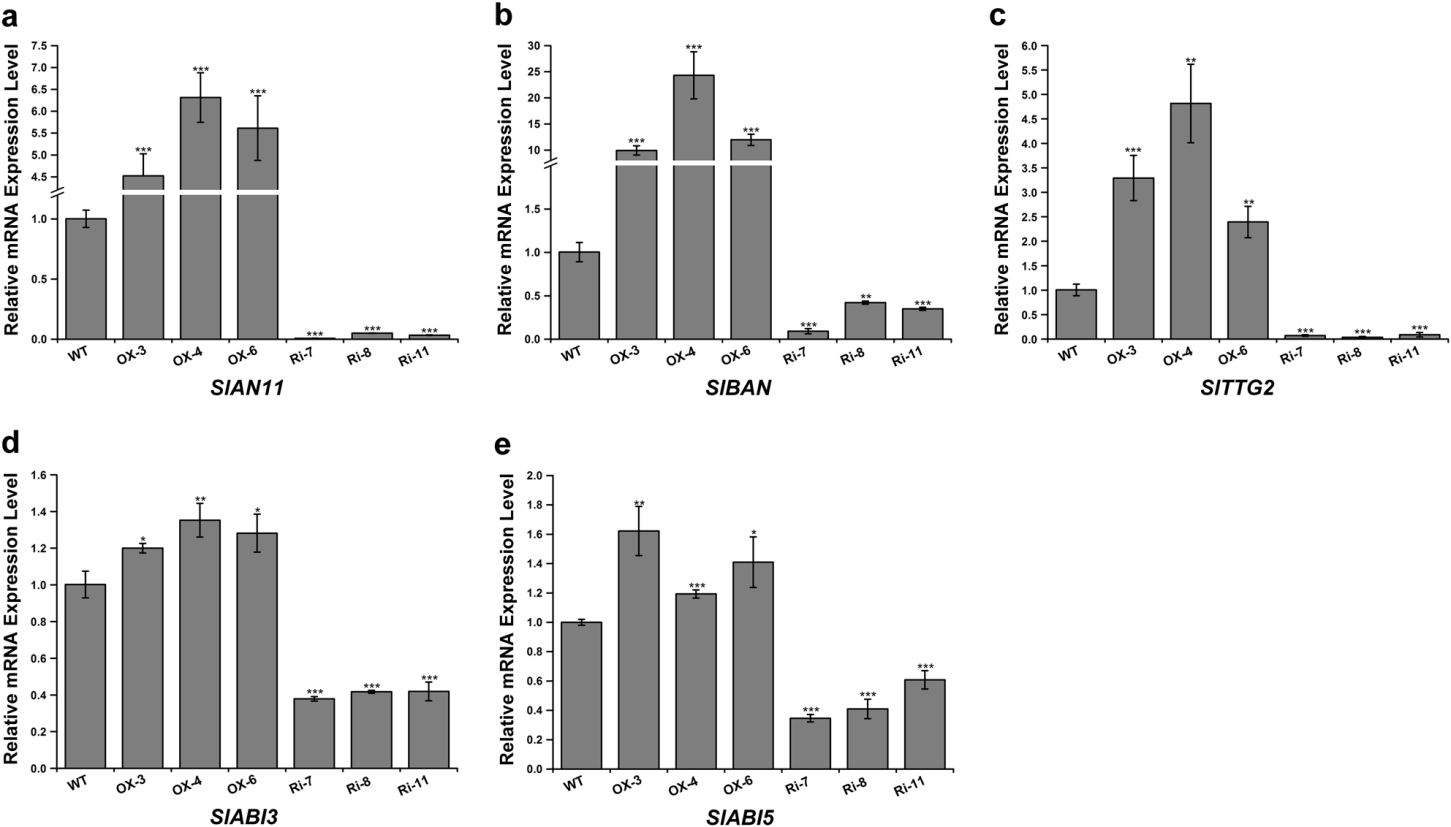
Expression levels analysis of genes regulating seed development and dormancy in wild-type and *SlAN11* transgenic seeds . Total RNAs were extracted from these mature seeds of WT, *35S::SlAN11* (lines OX-3, OX-4, and OX-5), and *SlAN11-RNAi* (lines Ri-7, Ri-8, and Ri-11) T2 homozygous transgenic plants. Quantitative real-time PCR analysis revealed the relative mRNA levels of *SlAN11* (**a**), *SlBAN* (**b**), *SlTTG2* (**c**), and ABA signal transduction genes *SlABI3* (**d**) and *SlABI5* (**e**). Results represent mean values ± SD from three biological replicates. Asterisks indicate statistically significant differences (**P < 0.01, *t*-test)

Anthocyanidin reductase encoded by the *BANYULS* (*BAN*) gene is the core enzyme in PA biosynthesis^37^. TTG1 can regulate *BAN* expression in the whole seed coat^38,39^. In addition, *Transparent Testa Glabra2* (*TTG2*) is also required for seed coat PA biosynthesis and acts downstream of TTG1/MYB/bHLH transcriptional complexes^34,40^. We therefore sought to determine whether overexpressing and downregulating *SlAN11* gene could affect transcriptional levels of these genes. First, the upregulated and downregulated transcripts of *SlAN11* in transgenic tomato seeds were confirmed by qRT-PCR (Fig. 9a). Figure 9b–c showed that the abundance of *SlBAN* and *SlTTG2* transcripts dramatically increased in *35S::SlAN11* transgenic plants seeds compared with WT. By contrast, their transcript levels were significantly downregulated in *35::SlAN11-RNAi* transgenic plants seeds. These results suggest that *SlAN11* controls PA biosynthesis in seed coat by regulating its downstream targets genes such as *SlBAN* and *SlTTG2*.

In addition, our pilot experiments observed that *35::SlAN11-RNAi* transgenic seeds exhibited higher germination rate than that of the WT and *35S::SlAN11* seeds. To explore the role of *SlAN11* in seed germination, WT, *35S::SlAN11*, and *35S::SlAN11-RNAi* seeds were assessed in terms of radicle emergence in absent or presence of various concentrations of ABA. Whether applying ABA or not, *35S::SlAN11-RNAi* transgenic seeds exhibited even higher germination rate and less sensitive to exogenous ABA than WT seeds during germination. By contrast, *35S::SlAN11* transgenic plants exhibited a certain degree of germination inhibition, more sensitive to ABA and reduced in radicle emergence in comparison with WT seeds (Fig. 8b). Upon increase of the exogenous ABA concentration, the germination of *35S::SlAN11* transgenic seeds was obviously inhibited with a sharper rate than WT seeds, but germination rate of the *35S::SlAN11-RNAi* seeds was reduced with a lower rate than WT seeds (Fig. 8b, d, e). In order to further understand the molecular mechanism of *SlAN11* in regulating seed germination, we detected the expression levels of *SlABI3* and *SlABI5* in the seeds of WT and *SlAN11* transgenic lines, as ABI3 and ABI5 are two major TFs in ABA signal transduction pathway and involved in ABA-dependent growth arrest during germination^31,41^. Compared with WT seeds, the abundance of *SlABI3* and *SlABI5* transcripts was significantly increased in the *35S::SlAN11*, whereas, their transcript levels were dramatically decreased in the seeds of *35S::SlAN11-RNAi* transgenic plants (Fig. 9d, e), suggesting that *SlABI3* and *SlABI5* were positively regulated by *SlAN11*.

### SlAN11 physically interacts with bHLH but not with MYB TFs

**Fig. 10:**
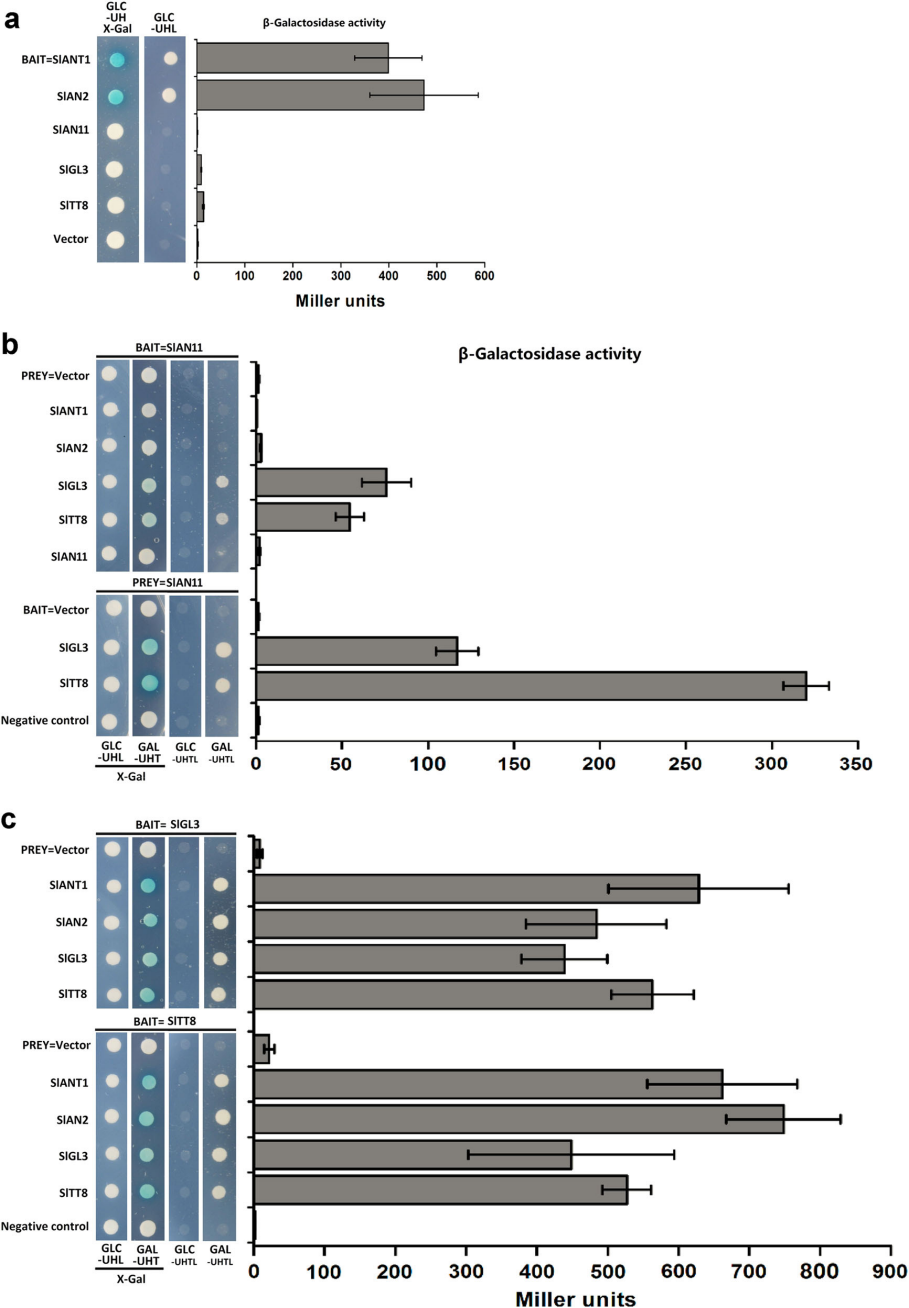
The interaction of SlAN11 with the bHLH TFs but not with the MYB TFs in yeast. **a** Transcriptional activity of SlAN11, SlANT1, SlAN2, SlGL3, and SlTT8 determined by the oNPG assay in yeast. Yeast strain EGY48 containing the pSH18-34 reporter plasmid was transformed pEG202-SlAN11, pEG202-SlANT1, pEG202-SlAN2, pEG202-SlGL3, and pEG202-SlTT8, respectively. Yeast colony grown on X-Gal plates were showed on the left panel with blue color indicating activation of *LacZα* marker gene by SlANT1 or SlAN2. **b** SlAN11 has interaction with SlGL3 and SlTT8 but not with SlANT1 and SlAN2. **c** bHLH TFs SlGL3 and SlTT8 interact with the MYB TFs SlANT1 and SlAN2, and can also homodimerize or heterodimerize with SlGL3 or SlTT8. The interaction was determined by the oNPG assay. Results represent mean values ± SD from three independent a-galactosidase assays

In *Arabidopsis*, anthocyanin biosynthesis is modulated by a regulatory complex containing WD40 proteins, MYB and bHLH TFs. We performed Y2H assays to determine whether SlAN11 can interact with the MYB TFs SlANT1 and SlAN2, as well as the bHLH TFs SlTT8 and SlGL3. We first tested the transcriptional activities of these TFs by yeast one-hybrid assay. Yeast colonies expressing SlANT1 and SlAN2 all exhibited strong auto-activation (Fig. 10a), as indicated by the blue color of yeast colonies on the X-Gal plates and growth on selection medium (–Ura–His–Leu). The quantitative analysis on the activity of β-galactosidase also proved SlANT1 and SlAN2 have strongly transcriptional potential. By contrast, SlAN11 and bHLH TFs SlTT8 and SlGL3 proteins did not show auto-activation (Fig. 10a).

Next, the Y2H system was constructed to assess the interaction between SlAN11 and the MYB TFs SlANT1 and SlAN2, as well as the bHLH TFs SlTT8 and SlGL3. The result showed that SlAN11 interacted with all of the bHLH TFs tested, but not with the MYB TFs SlANT1 and SlAN2 (Fig. 10b). The β-galactosidase activity also indicated that SlAN11 and the bHLH TFs SlTT8 and SlGL3 have strongly interactions but not MYB TFs.

In addition, we also tested whether the bHLH TFs SlTT8 and SlGL3 interacts with the MYB TFs SlANT1 and SlAN2. The results indicated that the MYB TFs SlANT1 and SlAN2 interacted with all of the bHLH TFs tested (Fig. 10c). Interestingly, bHLH proteins formed homodimers as well as heterodimers with SlTT8 or SlGL3. The interactions were also confirmed by quantification of β-galactosidase activity measurements. Taken together, our results suggest that the SlAN11 protein specifically interacts with bHLH but not with MYB TFs. Moreover, the bHLH TFs interacts with both SlAN11 and the MYB TFs, and can also homodimerize or heterodimerize with related bHLH proteins.

### Modification of SlAN11 alone does not affect anthocyanin biosynthesis in tomato fruits

**Fig. 11:**
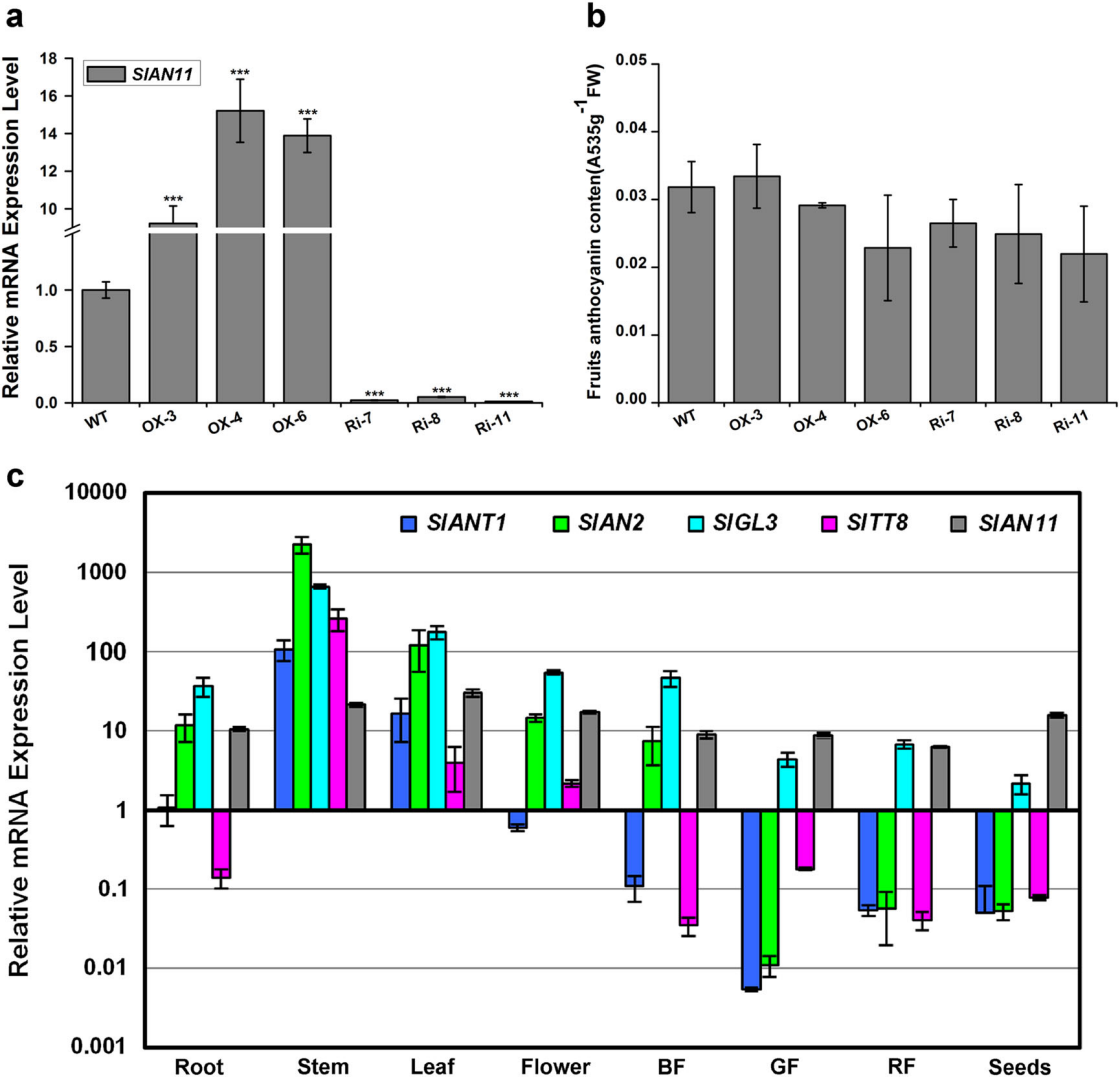
SlAN11 could not enhance anthocyanins a ccumulation in tomato fruits. **a** Expression level analysis of *SlAN11* in wild-type and *SlAN11* transgenic fruits. Total RNAs were extracted from these red fruit pericarps of WT, *35S::SlAN11* (lines OX-3, OX-4, and OX-5), and *SlAN11-RNAi* (lines Ri-7, Ri-8, and Ri-11) T2 homozygous transgenic plants. **b** Quantitative analysis of anthocyanin contents of red fruit pericarps from wild-type, *35S::SlAN11*, and *SlAN11-RNAi* transgenic lines. **c** The expression pattern analysis of *SlAN11*, *SlANT1*, *SlAN2*, *SlTT8*, and *SlGL3* in different tissues. Total RNAs were extracted from roots, stem, leaves, flowers, green fruit pericarps (GF), BF (breaker phase fruit), red fruit pericarps (RF), and mature seeds. Relevant mRNA levels were quantified by quantitative RT-PCR and are indicated as relative expression levels compared with the internal control *SlUBI3* mRNA. The vertical axis is log scale. Results represent mean values ± SD from three biological replicates. Asterisks indicate statistically significant differences ***P < 0.001; *t*-test)

The altered flavonoid compound accumulation in the leaves, stems, and seeds of *SlAN11* transgenic plants prompted us to investigate the effect of *SlAN11* on anthocyanin accumulation in tomato fruits. However, unlike the results with the vegetative tissues and seeds, overexpressing and downregulating *SlAN11* gene did not influence anthocyanin contents in tomato fruits (Fig. 11a, Supplementary Figure S4). Chemical analysis further confirmed these observations. Anthocyanin accumulation in fruits was not significantly altered in *35S::SlAN11* and *35S::SlAN11-RNAi* transgenic plants compared with WT (Fig. 11b). These results indicated that there exist other factors that function primarily in fruit tissues. In order to further determine the roles of the components of the MBW complex in anthocyanin production in tomato fruits, the expression levels of genes encoding WD40 protein (*SlAN11*), MYB TFs (*SlANT1* and *SlAN2*), and bHLH TFs (*SlTT8* and *SlGL3*) in different tissues of WT tomato plants was analyzed by using the qRT-PCR. As shown in Fig. 11c, unlike *SlAN11* and *SlGL3* genes, which were expressed in all tissues tested, the expression patterns of *SlANT1*, *SlAN2*, and *SlTT8* genes showed significant differences among tomato tissues. The transcripts of *SlANT1*, *SlAN2*, and *SlTT8* genes were mainly confined to roots (except *SlTT8*), stems, leaves, and flowers, while almost no expression was detected in pericarps of green fruits (except *SlAN2*), break fruits, red fruits, and seeds. Since anthocyanins normally do not accumulate in tomato fruits, our results suggested that the MYB TFs (SlANT1 and SlAN20, and the bHLH TF (SlTT8) may be important factors that affected the anthocyanin biosynthesis in tomato fruits.

## Discussion

### SlAN11 may modify flavonoid compound levels largely through the regulation of late genes in flavonoid biosynthesis

Anthocyanin biosynthesis is transcriptionally governed by the ternary MBW protein complex, which is constituted by one R2R3-MYB protein like PRODUCTION OF ANTHOCYANIN PIGMENTS 1/2 (PAP1/2), or MYB113/114, one bHLH protein like TT8, GLABROUS 3 (GL3), and one WD protein like TTG1. To date, some genes encoding WD40 protein have been characterized in different plant species, such as *Arabidopsis*^10^, apple^33^, strawberry^14^, and Chinese bayberry^42^. The above researches all suggest that WD40/TTG1 may play an important role in the biosynthesis of anthocyanins/PAs in plant. In our study, the *SlAN11* gene encoding a WD40 repeat protein in *S. lycopersicum* was identified with the function of regulating anthocyanin/PA biosynthesis by reverse genetics approach.

In *Arabidopsis*, the expression level of *TTG1* is indistinguishable throughout the plant^10^. In tomato, *SlAN11* is expressed either in the tissues of rich anthocyanins (leaf, stem, and seed) or in the tissues of less abundant anthocyanins (root, flower, and fruit), and its expression level is similar in all tissues, though highly expressed in leaves, stems, flowers, and seeds (Fig. 3a). These results were consistent with the results of histochemical GUS reporter assay (Fig. 3b–i). Under normal conditions, tomato mainly accumulates anthocyanins/PAs in stems, leaves, and seeds. So, its expression pattern is consistent with anthocyanin accumulation in these tissues, suggesting that *SlAN11* is involved in anthocyanin biosynthesis in tomato.

The *Arabidopsis ttg1* mutant lacks anthocyanins/PAs in all tissues, including the stems, leaves, and seeds^36,43^. The *SlAN11-RNAi* transgenic lines severely lack anthocyanins/PAs in leaves, stems, and seeds (Figs. 5 and 8, Supplementary Figure S2). In contrast, overexpression of *SlAN11* in WT tomato seedlings resulted in more anthocyanin accumulation in leaves and stems but not in tomato fruit than WT plants (Figs. 5 and 11b). In *Arabidopsis* *ttg1* mutant, whose anthocyanin biosynthesis is blocked at the DFR step, the transcript of upstream genes showed unaffected, like *CHS*, *CHI*, *F3’H*, and *FLS1*^36^. Similarly, the LBG *SlDFR* was remarkably downregulated in *SlAN11-RNAi* transgenic lines, and upregulated in *35S::SlAN11* transgenic lines while the transcription levels of EBGs *CHS*, *CHI*, and *F3’H* were unaffected in these transgenic lines (Fig. 6b–e). However, the transcript of *SlFLS1*, one EBG gene, was not affected by MBW complex in other model plant species, was remarkably decreased in *35S::SlAN11* transgenic plants, but not significantly changed in *SlAN11-RNAi* transgenic plants (Fig. 6f). Accordingly, flavonol staining analysis also revealed that the kaempferol and quercetin concentration all were significantly decreased in *35S::SlAN11* plants in comparison with the WT and *SlAN11-RNAi* transgenic plants (Fig. 7). These findings suggest that the high anthocyanin content in *35S::SlAN11* transgenic plants may result from modification of dihydroflavonol flow, increased flow to anthocyanins by improved *SlDFR* transcript but reduced flow to the accumulation of flavonol or its glycosylated derivatives by repressing the expression of *SlFLS*.

The accumulation of PA compounds in seeds is also regulated by MBW protein complexes^11,38^. In addition, downstream direct targets of MBW protein complexes, including other TFs such as the TTG2 WRKY protein, positively regulate the accumulation of PAs^34,40^. In particular, our results showed that the transcript abundance of the PA-specific gene *BAN* and the regulatory gene *SlTTG2* were strongly reduced in *35S::SlAN11-RNAi* transgenic seeds, which finally led to a deficiency of PAs in seeds (Fig. 8a, c). In contrast, their transcript levels were dramatically increased in *35S::SlAN11* transgenic seeds, which resulted in more PA accumulation in seed coats (Fig. 9b, c). Taken together, our data suggest a key role for *SlAN11* in the governing of seeds’ PA biosynthesis, with regulating directly its downstream targets genes *SlBAN* and *SlTTG2*.

In addition, TTG1 can also regulate *TT8* expression in *Arabidopsis* by taking part in the MYB–bHLH complexes interacting with the *TT8* promoter, and the activity of the *TT8* promoter is severely affected in *ttg1* mutant^44,45^. In our study, the abundance of *SlTT8* transcript significantly increased in the *35S::SlAN11* transgenic plants, and dramatically decreased in *35S::SlAN11-RNAi* transgenic plants (Supplementary Figure S5c), suggesting that *SlTT8* was positively regulated by SlAN11. However, the transcription levels of another bHLH gene *SlGL3* and MYB genes *SlANT1* and *SlAN2* were not significantly changed in these transgenic lines (Supplementary Figure S5). These findings suggest that SlAN11 influence anthocyanin biosynthesis in tomato not only by forming a MBW complex with SlTT8 but also by regulating the transcription of *SlTT8* gene.

### The function of SlAN11 in trichome formation

WD40 repeat proteins in *Arabidopsis* play a key role in trichome formation, but this function was not observed in some other plant species^46^. *Arabidopsis* *ttg1* mutant exhibited hairless phenotype in leaves and stems^10^. Besides glandular trichomes similar to *Arabidopsis* plants, tomato has non-glandular unicellular trichomes on stem and leaves^47,48^. Our study indicated that overexpression or RNAi of *SlAN11* gene didn’t affect trichome distribution on young leaves and stems (Supplementary Figure S3). Moreover, no *SlAN11*_*Pro*_*::GUS* activity was observed in trichome on the stems, leaves, and sepals (Fig. 3d, f, h). Our result was consistent with previous studies on *Medicago truncatula* and petunia. *MtWD40-1* mutations in *M. truncatula* didn’t pose impacts on the trichome development and distribution in leaves and petioles^13^. Modification of gene expression of AN11, a WD40 protein, also caused little effects in trichome phenotype in petunia^9^.

Like *AN11* in petunia and *PAC1* in maize, *SlAN11* is a single-copy gene, because no other *SlAN11* gene loci were recovered in *S. lycopersicum* genome databases by BLASTN analysis with the *SlAN11* nucleotide sequence as query. In addition, two new WD40 repeat proteins with more than 60% identity were recovered when BLASTP query was conducted using deduced amino-acid sequence of *SlAN11*. SlAN11-like protein (GenBank accession: XP_004235027) was 77% identical to SlAN11 at the amino-acid level, and another WD40 protein LWD1 (GenBank accession: XP_004238433) showed 61% identity to SlAN11. Therefore, it is possible that the lack of trichome change in the *SlAN11* overexpression and RNAi transgenic lines is due to genetic redundancy.

### SlAN11 regulates seed dormancy and cross talk with ABA signaling

During embryogenesis, *TTG1* not only affects biosynthesis of PAs but also affects seed dormancy. In *Arabidopsis*, the *ttg1* mutant reduced seed dormancy, as ascertained by a lower requirement for after-ripening and a higher germination rate^49^. In our study, *35::SlAN11-RNAi* transgenic seeds with transparent testa exhibited reduced dormancy and higher germination rate than WT seeds, whereas *35S::SlAN11* transgenic seeds with more PA accumulation in seed coat exhibited a certain degree of germination inhibition (Fig. 8). Some studies have shown that seed coat pigmentation was positively correlated with seed dormancy in crop plants. For example, red seeds of charlock (*Sinapis arvensis* L.) exhibit a reduced dormancy compared with black seeds^50^. In legumes, white seeds imbibe more rapidly than colored seeds and then germinate earlier. White seeds also suffer greater imbibition damage, as measured by higher solute leakage, which affects their vigor and viability^51,52^. In *Arabidopsis*, the reduced dormancy of testa mutants (such as *tt* mutants and *ttg1*) is closely related to increasing water penetration and a reduced thickness of the testa^49^. In legumes, water-impermeable seed dormancy was extensively studied and has been attributed to the presence of flavonoid compounds in the seed coat^52^. In tomato, the absence of condensed tannins in the three *anthocyaninsless* mutant (*ah*, *aw*, and *bls*) seeds contribute to increased water permeability and the rapid water uptake and germination^53^. Therefore, it is possible that the permeability and thickness of the testa are affected by the flavonoid compounds (PAs) and structural elements altered in the mutants, which may lead to effects on germination.

The phytohormone ABA plays a vital role in promoting seed dormancy and inhibiting seed germination^54^. *35::SlAN11-RNAi* transgenic seeds exhibited less sensitive to exogenous ABA and germinated earlier than WT seeds, in contrast, much more severe inhibition on the ABA-dependent germination was observed in the *35S::SlAN11* transgenic seeds. These results indicated that SlAN11 is a key factor that can both control PA contents in seed coats and regulate seed dormancy and germination in an ABA-dependent way. In ABA signal transduction pathway, Abscisic Acid Insensitive3 (ABI3) and ABI5 are two major TFs and execute ABA-dependent growth arrest during germination^31,41^. The *abi3/vp1 and abi5* mutant seeds are desiccation-intolerant, insensitive to ABA, and usually germinate precociously in *Arabidopsis* and maize^54,55^. In addition, Chen’s data suggested that abundance of *ABI3* transcript was upregulated in developing seeds of *Arabidopsis* *ttg1* mutant^56^. Interestingly, the abundance of *SlABI3* and *SlABI5* significantly dramatically decreased in *SlAN11-RNAi* plants compared with WT seeds, whereas, their transcript levels were increased in the *35S::SlAN11* transgenic plants (Fig. 9d, e). These results suggested that *SlABI3* and *SlABI5* were positively regulated by the *SlAN11*. In *Arabidopsis*, a study indicated that PAs could inhibit seed germination by sustaining a high level of ABA contents^57^. Taken together, our results provided molecular evidences to illuminate how *SlAN11* regulates seed dormancy in tomato through ABA signaling pathway.

### SlAN11-dependent MBW complex is required for anthocyanin accumulation in tomato fruits

WD40 is a crucial component of the MBM protein complex. In *Arabidopsis*, TTG1 protein interacts with both the bHLH TFs (TT8 and GL3) and the MYB TF (TT2) to regulate the expression of anthocyanin and PA biosynthetic genes^45^. However, in most other plants studied, the WD40 protein only interacts with the bHLH TFs and not with the MYB TFs^33,58,59^. Y2H assays proved that SlAN11 interacts only with bHLH TFs in tomato, including SlTT8 and SlGL3 (Fig. 10b). Interestingly, bHLH TFs, including SlTT8 and SlGL3 were found to interact with SlAN11, as well as with MYB TFs SlANT1 and SlAN2, and could also homodimerize or heterodimerize with SlTT8 or SlGL3 protein (Fig. 10c). These results were similar to those of other recent observations and studies^33,60^.

Recent research indicated high positive correlation between the anthocyanin accumulation and the expression level of *WD40* in pomegranate fruit^3^. However, no remarkable alterations in anthocyanin contents were discovered in *35S::SlAN11* or *35S::SlAN11-RNAi* transgenic tomato fruits (Fig. 11b, Supplementary Figure S4). The result leads to the conclusion that SlAN11 alone could not enhance anthocyanin biosynthesis in tomato fruits. So what caused the absence of anthocyanin accumulation in tomato fruits? It is known that a complex containing a MYB protein, bHLH, and WD40 TF (MBW complex) is necessary for the appropriate regulation of the biosynthesis of anthocyanins in various plant species. Therefore, we examined the expression patterns of *SlAN11*-, *SlANT1*-, and *SlAN2*-encoded MYB TF, as well as *SlTT8*- and *SlGL3*-encoded bHLH TF in different tissues of WT tomato. Interestingly, *SlANT1*, *SlAN2*, and *SlTT8* transcripts were mainly confined to root (except *SlTT8*), stems, leaves, and flowers, with almost no expression in green fruit (except *SlAN2*), break fruits, red fruits, and seeds (Fig. 11c). By contrast, like *SlAN11*, *SlGL3* was expressed ubiquitously in all organs examined. Our results implied that the MYB TFs (SlANT1 and SlAN2) and the other bHLH TF (SlTT8) may be important factors that affected the anthocyanin biosynthesis in tomato fruits. In addition, some studies have also shown that the overexpression of *SlANT1* or *AtMYB75/PAP1* in tomato resulted in high levels of anthocyanins in their vegetative tissues, but limited purple coloring on pericarp of tomato fruits^18,61^. Overexpression of the *SlAN2* in tomato led to the same phenotype in vegetative tissues as *SlANT1*, but unexpectedly, no anthocyanin accumulation was noted in tomato fruits^62^. Heterologous expression of *BoPAP1* in tomato induced stamen-specific anthocyanin accumulation, and observed the consistency of *SlTT8* expression and anthocyanin accumulation in purple stamens^63^. Together, these results lead to the conclusion that low expression of *SlANT1*, *SlAN2*, and *SlTT8* in tomato fruits might be responsible for the low accumulation of anthocyanins in WT and *SlAN11* overexpression fruits, and modification of only one MBW member, like *SlAN11*, *SlTT8*, *SlANT1*, and *SlAN2*, causes little effects in the fruit anthocyanin contents. Therefore, we speculated that co-expression of *MYB* and *bHLH* genes may be an optimized transgenic strategy for improving anthocyanins accumulation in tomato fruits.

**Fig. 12:**
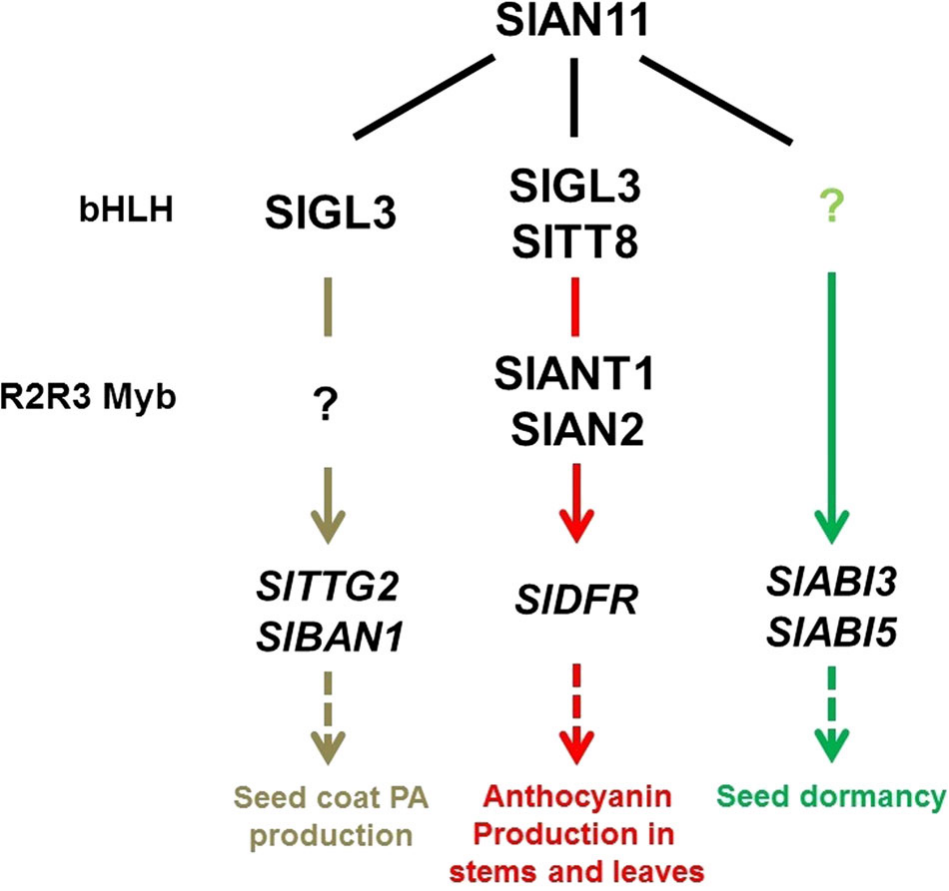
The model for SlAN11-dependent regulation of anthocyanin/PA accumulation and seed dormancy. Solid lines indicate interactions between members of a complex. Solid arrows indicate positive regulations. Dashed arrows indicate a multi-step differentiation pathway. Colored lines and arrows indicate specific regulator combinations and the pathway controlled

On the basis of our results and previous studies in model plant species, we proposed a hypothetical working model for SlAN11-dependent regulation of anthocyanin/PA accumulation and seed dormancy (Fig. 12). In tomato stems and leaves, SlAN11 interacts with bHLH TFs (SlTT8 and SlGL3), and forms a MBW complex with MYB (SlANT1 and SlAN2) and bHLH TFs to regulate the transcription of *SlDFR* gene to control anthocyanin biosynthesis. In addition, for the absence of SlANT1, SlAN2, and SlTT8 in tomato seeds, SlAN11 may interact with SlGL3 or other unknown bHLH TFs, and forms a MBW complex with unknown R2R3 Mybs and bHLH TFs to regulate the transcription of both *SlTTG2* and *SlBAN* genes to regulate PA biosynthesis in seed coats. Moreover, SlAN11 may participate in a complex with other proteins involved in seed dormancy to regulate the transcription of *SlABI3* and *SlABI5* genes to influence seed dormancy. However, the proteins that can interact with SlAN11 in tomato seeds are still unknown and require further investigation.

## Electronic supplementary material

Supplementary Tables 1-3+Figures 1-5
