# Supplementary material for: Tomato SlAN11 regulates flavonoid biosynthesis and seed dormancy by interaction with bHLH proteins but not with MYB proteins
Source: Hortic Res. 2018 Jun 1;5:27. doi: 10.1038/s41438-018-0032-3 (PMC5981465; doi:10.1038/s41438-018-0032-3)
Supplement: Supplementary file 1 — Supplementary Tables 1-3+Figures 1-5 [file 41438_2018_32_MOESM1_ESM.pdf]

Supplementary Table 1 Primer sequences of *SlAN11* gene used in for construction vectors.

Gene	Forward primer (5′→3′)	Reverse primer (5′→3′)	Vector type
SlAN11	TCTCTAGAATGGAGAATTC AAGTCAAGAATC	GAGCTCAAAACTCAATTTA TCCCATTCG-3	Overexpression
SlAN11	GAGCTCGTCTGTTTCTGCT GATGGATC	GAATTCCTCTACCAAAGAA AGACAGCC	RNAi
SlAN11	CTCGAGTCTAGAGTCTGTT TCTGCTGATGGATC	AAGCTTCTCTACCAAAGAA AGACAGCC	RNAi
SlAN11	TCTAGAATGGAGAATTCAA GTCAAGAA	CTCGAGTACTTTAAGCAGC TGCAACT	GFP
SlAN11 Pro	GGTACCTGCTACTAATTTTG TGAGTGAAG	TCTAGAATTTCTACTAGAGA TTATAATTGAAATG	GUS

Supplementary Table 2 Primer sequences of related genes used in quantitative real-time PCR

Gene	Forward primer (5′→3′)	Reverse primer (5′→3′)
SlCHI	TCAAGGCTTCCAGGATATGTCC	GGATGTCCCGAACTTCTCCTTG
SlCHS	GGTAGCATAAGGGTAGCAGCC	CTTAAATGGAGGCAAAGCACTT
SlF3'H	GGCGGAAAACTTACTGATACAGA	TGACTGTTGGTTTGATTACTTCG
SlDFR	CCAG GTCAGATTCCATAACGAG	TGACTGTTGGTTTGATTACTTCG
SlFLS	CAATGGAGGTAGCAAGGGTC	TGACTGTTGGTTTGATTACTTCG
SlBAN	ATCTCTCCTGGCTGGTCCTTAC	TGGGCTCGACAAACATCCTC
SlTTG2	CTCATCCACAAATGTTCGTTCTG	CCTTGGGTTTCTCTACCGATTG
SlAN11	ATTGGAATGAAGTGGAGCCG	CCATCAGCAGAAACAGACGC
SlABI3	ATTGGGACTTGTCGTGTTTGG	CCACACTTTATGTCGGCGTAT
SlABI5	GTAGAATGGGAAACGGTAGCG	ATCCGACGACACGGGACTTAG
SlUBI3	AGGTTGATGACACTGGAAAGGTT	AATCGCCTCCAGCCTTGTTGTA
SlANT1	GAAAAGTTGTAGATTGAGGTGGC	CCAATAGTTTTTCACATCGTTAGC
SlAN2	ATCGTTGGGAGTTAGGAAAGG	GATAGTTTAGCCACCTTAGTCTGC
SlGL3	GCCAAGGGTTACCAGGGA	GAGGTTAGGATCTTCCGTGACA
SlTT8	TTCCTCTCTTGGACGGTGTTG	CCGAAGTGGAGTGCTCAGATAAA

Supplementary Table 3 Primer sequences of related genes used in Yeast one/two-hybrid assays

Gene	Forward primer (5′→3′)	Reverse primer (5′→3′)
SlAN11	CCGCGGATGGAGAATTCAAGTCAAGAA	CTCGAGTTATACTTTAAGCAGCTGCAA
SlANT1	TCTAGAGAATTCATGAACAGTACATCTATGTCTTCA	CTCGAGTTAATCAAGTAGATTCCATAAGTC
SlAN2	TCTAGAGAATTCATGAATACTCCTATGTGTGCATC	CTCGAGCTCTAATATAAATATCAAACGTCG
SlGL3	GGATCCCCGCGGATGGCTATGGGACACCAA	CTCGAGGACCAACCTTGACTGACTAATC
SlTT8	TCTAGAGAATTCATGGAGATTATACAGCCTAATAGC	CTCGAGGAAAGAGCTGAGCAAATTATAG

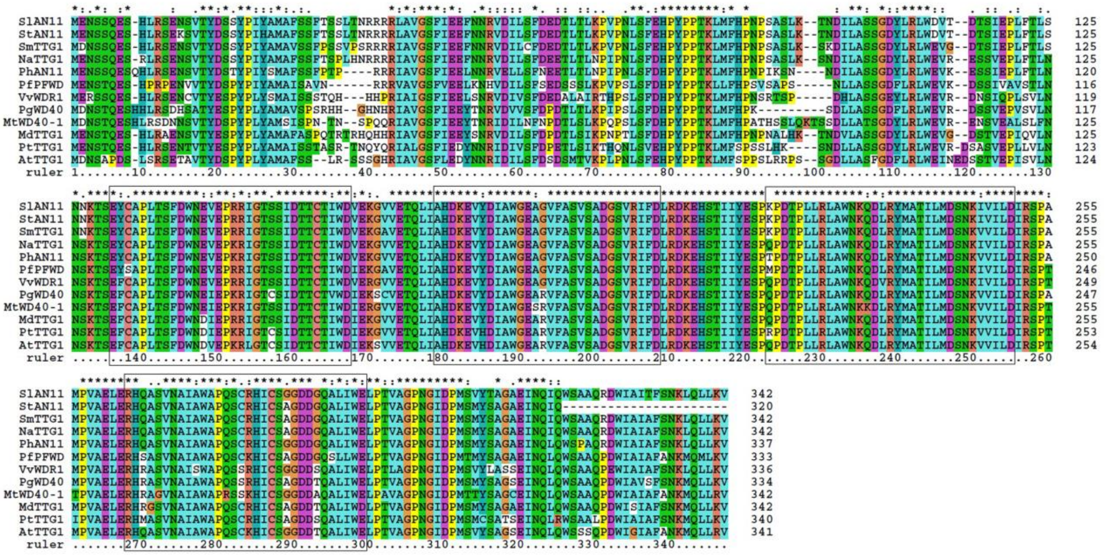

Supplementary Figure S1 Alignment of deduced amino acid sequences of plant homologues to the Arabidopsis TTG1/WD40 protein.

The WD40 repeat domains are indicated with open boxes. The name of the proteins, their source and GenBank accession numbers are as follows: SlAN11 isolated from *Solanum lycopersicum* (tomato) (XP_004235332); SpTTG1-like from *Solanum pennellii* (XP_015067455); StAN11 from *Solanum tuberosum* (potato) (AEF01097); SmTTG1 from *Solanum melongena* (AJN91103); PhAN11 from *Petunia x hybrid* (AAC18914); PtTTG1 from *Populus trichocarpa* (XP_002318500); MtWD40-1 from *Medicago truncatula* (XP_003602392); PfPFWD from *Perilla frutescents* (BAB58883); MdTTG1-like from *Malus domestica* (NP_001315674); AtTTG1 from *Arabidopsis thaliana* (CAC10524), NtTTG1 from *Nicotiana tabacum* (ACJ06978); PgWD40 from *Punica granatum* (ADV40946); VvWDR1 from *Vitis vinifera* (NP_001268101); NaTTG1 from *Nicotiana attenuate* (OIT04713); IpWDR from *Ipomoea purpurea* (ABW69689); SiTTG1 from *Sesamum indicum* (XP_011075888); RiTTG1 from *Rubu sidaeus* (AEI55401); FaTTG1 from *Fragaria x ananassa* (AFL02466); MnTTG1 from *Morus notabilis* (XP_010099415).

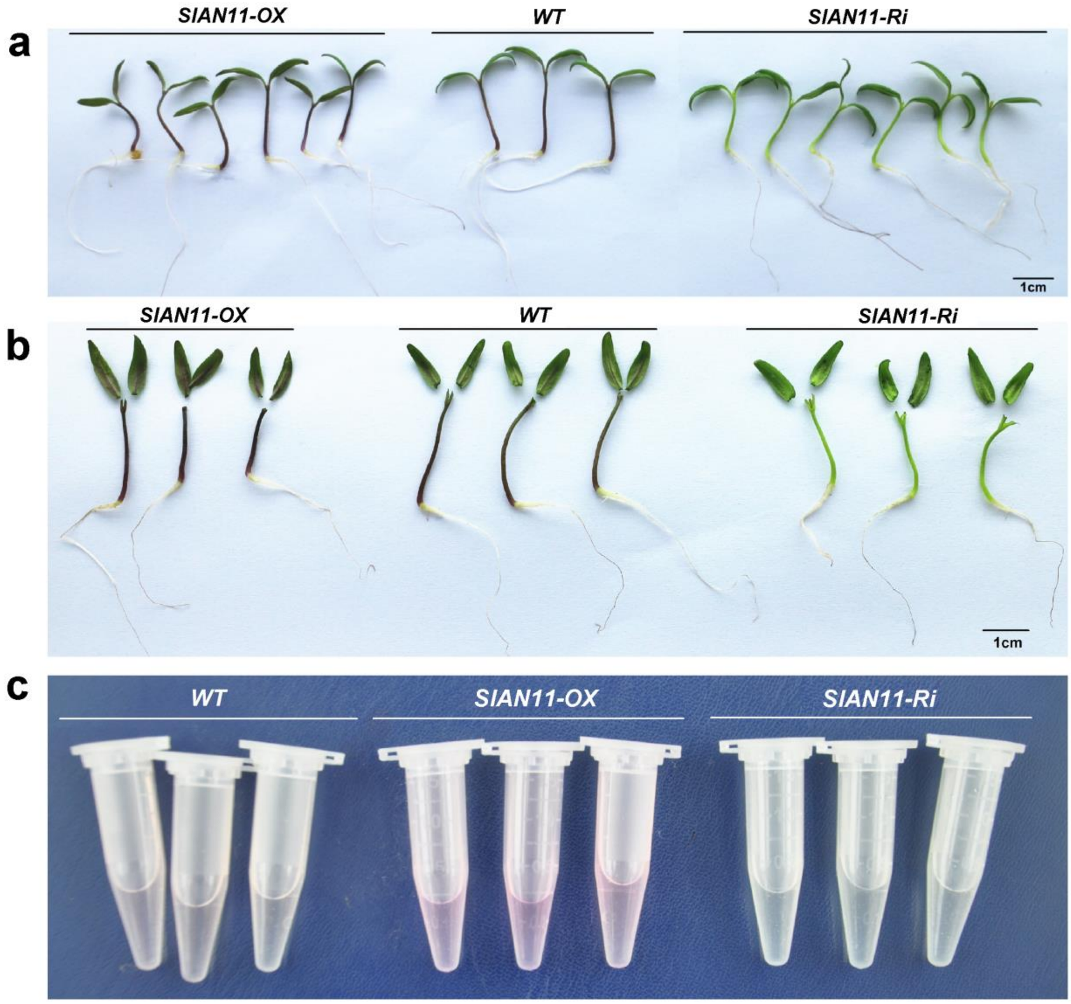

Supplementary Figure S2 Phenotypes of anthocyanins accumulation of wildtype and *SlAN11* transgenic seedlings

(**a, b**) Phenotypes of anthocyanins accumulation in cotyledons and hypocotyl from wild-type (*WT*), *35S::SlAN11* (*SlAN11-OX*) and *SlAN11-RNAi* (*SlAN11-Ri*) transgenic seedlings. (**c**) Extracts of anthocyanins from 5-days old seedlings. The seedlings were grown in 1/2MS culture bottle and observed 5 days after germination.

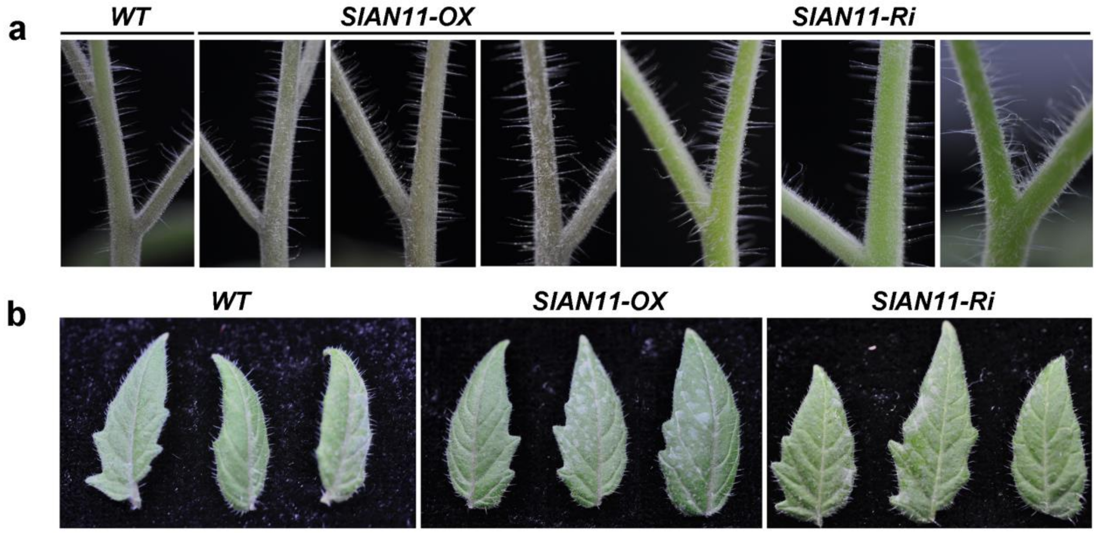

Supplementary Figure S3 SlAN11 does not affect trichome development in tomato plants

(**a, b**) Trichomes of stems and leaves from 30d-old wild-type (WT), *35S::SlAN11* (*SlAN11*-*OX*) and *SlAN11*-*RNAi* (*SlAN11*-*Ri*) transgenic plants.

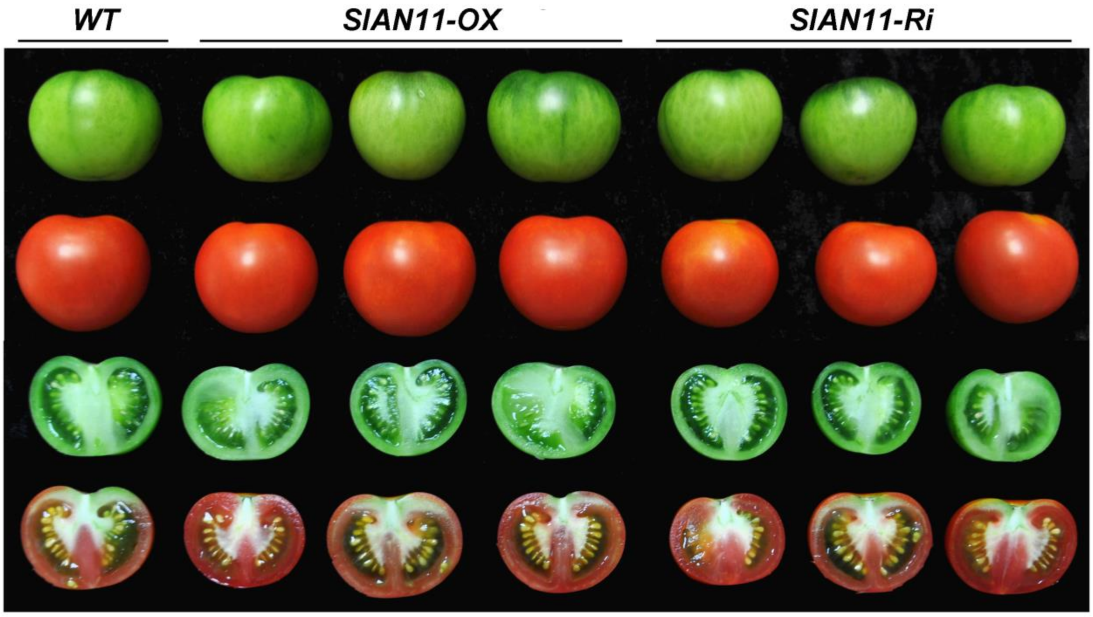

Supplementary Figure S4 No anthocyanins accumulation in *SlAN11* transgenic tomato fruits.

Phenotype of *35S::SlAN11* and *SlAN11-RNAi* lines compared to the control non-transformed plants (Ailsa Craig). Details of immature green fruits, section of the immature green fruits, red ripened fruits and section of the red fruits are shown.

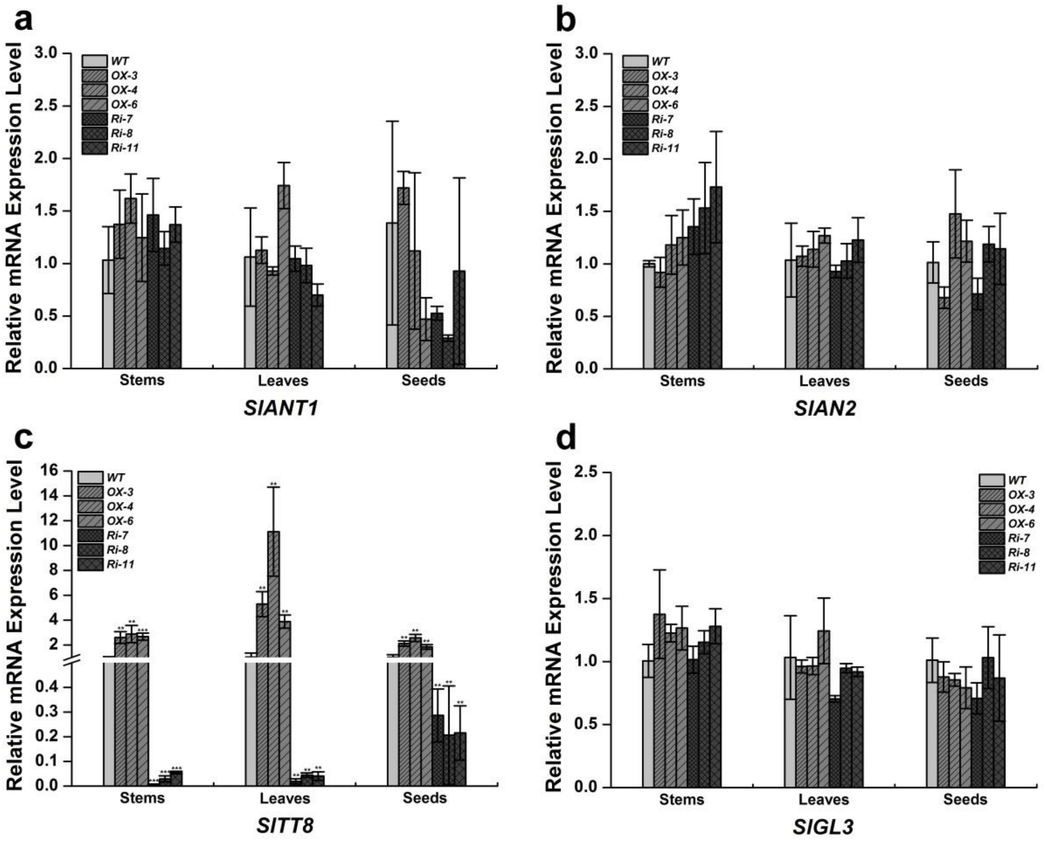

Supplementary Figure S5 Expression levels analysis of the endogenous *bHLH* and *MYB* genes in stems, leaves and seeds of wildtype and *SlAN11* transgenic lines.

Total RNAs were extracted from stems, leaves and mature seeds. Relevant mRNA levels were quantified by quantitative RT-PCR and are indicated as relative expression levels compared with the internal control *SlUBI3* mRNA. Results represent mean values ± SD from three biological replicates. Asterisks indicate statistically significant differences (*P<0.05, **P<0.01, ***P<0.001; *t*-test).
